# Genome-wide analysis of macrosatellite repeat copy number variation in worldwide populations: evidence for differences and commonalities in size distributions and size restrictions

**DOI:** 10.1186/1471-2164-14-143

**Published:** 2013-03-04

**Authors:** Mireille Schaap, Richard JLF Lemmers, Roel Maassen, Patrick J van der Vliet, Lennart F Hoogerheide, Herman K van Dijk, Nalan Baştürk, Peter de Knijff, Silvère M van der Maarel

**Affiliations:** 1 Department of Human Genetics, Leiden University Medical Center, Leiden, The Netherlands; 2 Department of Econometrics & Tinbergen Institute, Vrije Universiteit Amsterdam, Amsterdam, The Netherlands; 3 Econometric Institute & Tinbergen Institute, Erasmus University Rotterdam & Vrije Universiteit Amsterdam, Amsterdam, The Netherlands; 4 Econometric Institute, Erasmus University Rotterdam, Rotterdam, The Netherlands; 5 The Rimini Centre for Economic Analysis, Rimini, Italy

**Keywords:** Tandem repeat sequences, DNA copy number variations, Population genetics, Bayes theorem

## Abstract

**Background:**

Macrosatellite repeats (MSRs), usually spanning hundreds of kilobases of genomic DNA, comprise a significant proportion of the human genome. Because of their highly polymorphic nature, MSRs represent an extreme example of copy number variation, but their structure and function is largely understudied. Here, we describe a detailed study of six autosomal and two X chromosomal MSRs among 270 HapMap individuals from Central Europe, Asia and Africa. Copy number variation, stability and genetic heterogeneity of the autosomal macrosatellite repeats RS447 (chromosome 4p), MSR5p (5p), FLJ40296 (13q), RNU2 (17q) and D4Z4 (4q and 10q) and X chromosomal DXZ4 and CT47 were investigated.

**Results:**

Repeat array size distribution analysis shows that all of these MSRs are highly polymorphic with the most genetic variation among Africans and the least among Asians. A mitotic mutation rate of 0.4-2.2% was observed, exceeding meiotic mutation rates and possibly explaining the large size variability found for these MSRs. By means of a novel Bayesian approach, statistical support for a distinct multimodal rather than a uniform allele size distribution was detected in seven out of eight MSRs, with evidence for equidistant intervals between the modes.

**Conclusions:**

The multimodal distributions with evidence for equidistant intervals, in combination with the observation of MSR-specific constraints on minimum array size, suggest that MSRs are limited in their configurations and that deviations thereof may cause disease, as is the case for facioscapulohumeral muscular dystrophy. However, at present we cannot exclude that there are mechanistic constraints for MSRs that are not directly disease-related. This study represents the first comprehensive study of MSRs in different human populations by applying novel statistical methods and identifies commonalities and differences in their organization and function in the human genome.

## Background

More than half of the human genome consists of repetitive DNA and tandemly repeated DNA sequences comprise a significant proportion thereof. Based on the size of the individual repeat units and the total array size these tandem arrays are classified as micro-, mini-, macro- and megasatellite repeats. Microsatellites or short tandem repeats (STRs) for example often contain units of 1-7 base pairs (bp) and can span up to 600 bp. These small repetitive sequences make up a relatively small part of the human genome that has been intensively studied. At the other side of the repeat array spectrum, macrosatellite repeats (MSRs) consist of repetitive units of minimally 100 bp but are typically several kilobases (kb) large. MSRs have recently attained more attention because of their potential structural and regulatory role in the human genome. MSRs are now recognized to embody an extreme example of copy number variation, often spanning hundreds of kilobases of genomic DNA. A recent survey identified a large number of known and unknown MSRs distributed over the entire human genome [1].

Some MSRs, such as the macrosatellite repeat D4Z4 (3.3 kb unit) in the subtelomere of chromosome 4q, have already been studied in more detail. In the general population the D4Z4 (4q) MSR varies between 11-100 copies of the D4Z4 monomer. Reduction of the D4Z4 (4q) repeat array below the size of 11 units creates a more open D4Z4 chromatin structure that is thought to cause facioscapulohumeral muscular dystrophy (FSHD [MIM:158900]), an autosomal dominant disease characterized by progressive wasting and weakness of the facial-, shoulder and upper arm muscles [2-4]. In the subtelomere of chromosome 10q an almost identical MSR is located, but contractions of this D4Z4 (10q) repeat array are usually not associated with disease [5]. On the X chromosome the MSR DXZ4, with a unit size of 3 kb, was shown to vary between 12-100 copies in a study of 22 individuals [6]. Its polymorphic character is further underscored with an observed meiotic instability of 8.3% in three CEPH families [6]. Chromatin studies have shown that DXZ4 has opposite chromatin configurations on the active and inactive X chromosome. It is bidirectionally transcribed producing long non-coding RNAs and small RNAs. Based on these observations this MSR was suggested to play a role in the establishment or maintenance of X chromosome inactivation [7].

Some MSRs encode for a protein-coding gene in every repeat unit. D4Z4 for example encodes for the double homeobox protein 4 (DUX4), a putative germ line transcription factor [8]. Other MSRs, like DXZ4, are thought to produce non-coding RNAs which may either be important for the establishment and maintenance of the MSR chromatin structure, or may have other functionalities [7]. Therefore, there is a distinct possibility that natural size variation of MSRs can affect gene expression in cis and in trans. As is the case for D4Z4, where repeat contraction inflicted chromatin changes result in the loss of control over the *DUX4* gene, it is not inconceivable that inappropriate transcriptional activity of any MSR may have phenotypic consequences, including disease. Indeed, we and others have shown that the chromatin structure and transcriptional activity of MSRs is tightly controlled and that in disease conditions, like FSHD and cancer, there is a loss of control over their regulation [4,9-11].

Some other MSRs have also been studied to various levels of detail. MSR RS447 on chromosome 4p16.1 is composed of units of 4.7 kb in size each encoding for a deubiquitinating enzyme [12]. In a study of 37 Japanese individuals the array size of RS447 ranged from 20-103 units with evidence for somatic mosaicism and meiotic instability comparable to that reported for DXZ4 [13]. MSR5p, also described as TAF11-like [14], on chromosome 5p15.1 was recently suggested to be involved in a psychiatric disorder [15]. No significant difference in repeat length distribution was observed between individuals of European and African ethnicity, based on a qPCR analysis of both alleles collectively in 789 individuals [15]. A Southern blot based analysis on 22 individuals showed that the repeat array varied between 10-98 repeat units [14]. Units of the MSR FLJ40296, also described as the PRR20 array [14], on chromosome 13q21.1 encode for a proline rich protein. In a small study of 11 individuals this repeat array was shown to vary in size between 5-20 units [14]. In close proximity to the *BRCA1* locus resides the RNU2 repeat array having 6.1 kb repeat units each encoding for a U2 small nuclear RNA. The array length varies between 30-250 kb [16]. Finally, CT47 is an X chromosomal MSR, consisting of 4.8 kb repeat units that encode for a cancer testis antigen [17]. Normally, expression of *CT47* is restricted to the germ line, but its transcriptional repression is lost during the oncogenic process in small cell lung carcinomas [11].

Some MSRs have been studied only in silico [1], others are studied more extensively by Southern blot or PCR analysis. Apart from D4Z4 [18] and MSR5p [15], there are no studies that have systematically interrogated repeat length variation of MSRs in different populations. In this study we present the results of combined wet-lab and bioinformatic analyses of eight different MSRs, mapped to different locations in the human genome (Table 1). Individuals from the HapMap panels representing three different populations were included in this study to investigate MSR behavior on the population level. We also applied novel powerful Bayesian statistical evaluation methods to analyze repeat size variation of individual MSRs and all MSRs collectively. This study thereby represents the first in depth collective analysis of MSRs in different populations to identify commonalities and differences in the genomic regulation of MSRs. Our study provides evidence that MSRs are large, highly polymorphic regions in our genome with multimodal size distributions and minimal array length constraints, independent of genomic localization, protein coding potential, and unit size.

**Table 1:** Overview of the MSRs studied

**Name**	**Locus (hg18)**	**Unit (kb)**	**Restriction enzyme**	**Encoded product**
RS447	4p16.1	4.7	*Bam*HI	deubiquitinating enzyme (USP17)
MSR5p	5p15.1	3.4	*Hin*dIII	RNA polymerase-like
FLJ40296	13q21.1	6.6	*Hin*dIII	proline rich protein 20
RNU2	17q21-22	6.1	*Eco*RI	non-coding U2 small nuclear RNA
DXZ4	Xq23	3.0	*Eco*RI	long non-coding RNA
CT47	Xq24	4.8	*Eco*RI	cancer testis antigen 47
D4Z4 (4q)	4q35.2	3.3	EH/EB/X^a^	DUX4
D4Z4 (10q)	10q26.3	3.3	EH/EB/X^a^	DUX4-like

^a^EH/EB/X = *Eco*RI + *Hin*dIII/*Eco*RI + *Bln*I/*Xap*I.

## Results

**Figure 1:**
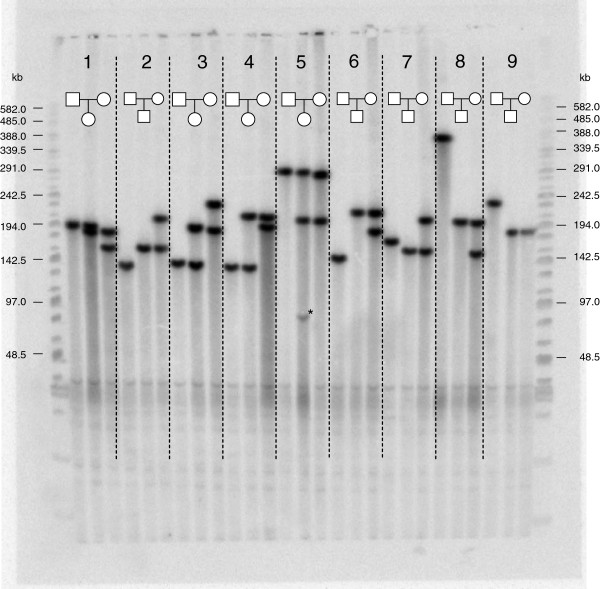
**Pulsed-field gel electrophoresis for DXZ4.** Southern blot analysis of DNA from 27 individuals from the African population, digested with *Eco*RI and separated by pulsed-field gel electrophoresis, and hybridized with the probe for the X chromosomal MSR DXZ4. The vertical lines distinguish the nine father-child-mother trios. Trio 5 (family Y077) shows the daughter inheriting her longest DXZ4 array (288 kb – 93 units) from father and the maternal X chromosome containing a shorter array (202 kb – 64 units). Besides the two parental arrays, a third fragment (asterisk) with lower signal intensity was observed, indicating a mitotic instability of the DXZ4 repeat leading to mosaicism where 8% of the cells contained a shorter DXZ4 array (86 kb – 25 units) than those inherited from the parents. Trio 8 (family Y071) illustrates that DXZ4 is a very polymorphic MSR, where the father had a DXZ4 array composed of 120 units and the mother two DXZ4 arrays of 63 and 48 units, respectively. The marker is indicated on the left and right side of the blot.

After PFGE-based repeat array sizing (see for example Figure 1), bioinformatic analyses were performed on the size of the repeat array of eight different MSRs in 210 unrelated individuals of Caucasian, Asian and African origin. Additional parameters that were investigated include genetic heterogeneity, mitotic and meiotic instability, and multimodality. Mosaic or complex repeat arrays were excluded from most analyses and only included in investigations of mitotic and meiotic instability.

For every individual the repeat size obtained by PFGE analysis was recalculated into the total number of repeat units (Additional file 1). The size distributions showed no significant gender difference for any of the autosomal repeats and DXZ4 (0.124 < p < 0.724), therefore all unrelated individuals for each population were pooled. For the X chromosomal MSR CT47 however, a p-value of 0.010 suggested that men have a different size distribution compared to women. A detailed inspection of the populations separately showed that this difference was observed in the Asians (p < 0.001) but not in the Caucasian (p = 0.697) or African (p = 0.760) population. In the Asian population male individuals had on average arrays consisting of 5.7 units compared to 6.9 units in female individuals, however, the total size variation was very small (middle 90% range of data: 4-8 units in males vs. 4-10 units in females). Therefore, also for CT47 we analyzed all unrelated individuals collectively. Because the Caucasian and African populations consisted of father-mother-child-trios, the repeat lengths of the children were used as controls for our sizing method. No differences in length between parents and child were found, except for two individuals having a meiotic contraction or expansion, indicating that our sizing measurements were accurate.

### Genetic heterogeneity

**Figure 2:**
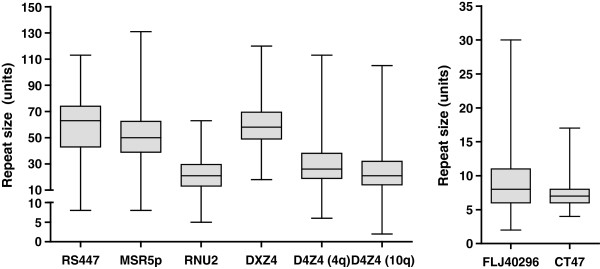
**Observed size ranges of the MSR arrays.** For every MSR, a box plot illustrates the size range observed in all 210 unrelated individuals. The box indicates the upper and lower quartiles and the median is represented by a horizontal line within the box. The vertical lines show the full range of sizes observed for the MSRs. MSRs are very polymorphic with some showing a wide range of size variation (e.g. MSR5p) and others a much more limited variation (e.g. CT47). The mean repeat array size was also very variable; e.g. RS447 repeats contained on average 60 repeat units in contrast to e.g. FLJ40296 that consisted on average of 9 repeat units. For FLJ40296, RNU2, CT47, D4Z4 (4q) and D4Z4 (10q) MSR array sizes were not evenly distributed where skewness to the longer repeat sizes was observed. A minimum number of two repeat units is required to form a repeat array and this was found for D4Z4 (10q) and FLJ40296, but for the other MSRs higher minimum array sizes were observed. This minimum number of repeat units depended on the MSR and could be rather large, e.g. for RS447 and MSR5p where the shortest repeat array consisted of eight units.

**Figure 3:**
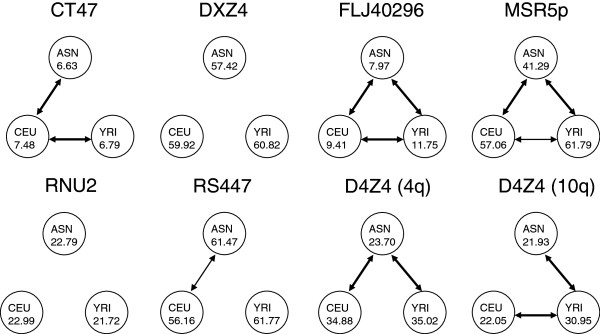
**Results of genetic heterogeneity test by comparing the means of the populations.** Genetic heterogeneity was investigated by cumulative distribution functions and mean repeat array size of the MSRs for each population. The results of the genetic heterogeneity test by comparing the means of the populations are illustrated for each MSR. The populations are depicted as a circle describing their mean repeat array sizes in number of repeat units. Dashed lines indicate a significant difference between populations (p-value < 0.05) and solid lines a p-value < 0.01. Both RNU2 and DXZ4 showed similar means of repeat array sizes in the different populations, while the means of the other six MSRs were significantly different for at least two populations. Genetic heterogeneity was variable for the MSRs, where it depended on the MSR which populations showed similar mean array sizes or significant differences.

MSR sizes showed a wide range of variation confirming and extending previous reports that MSRs are very large polymorphic structures in the human genome (Figure 2, Additional file 2: Table S2). Except for FLJ40296 and D4Z4 (10q), a minimum number of more than two repeat units was observed for each of the MSRs. Only for MSR5p in the African population (p = 0.838) and for DXZ4 in the Caucasian population (p = 0.175) MSR sizes were normally distributed according to the Shapiro-Wilk test. All MSRs in the Asian population were significantly skewed to longer repeat sizes and showed significant (excess) kurtosis, where repeat sizes were more concentrated around the mean leading to more peaked distributions compared to a normal distribution, except RS447. In Caucasians the autosomal MSRs demonstrated both skewness and (excess) kurtosis, although the skewness was not significant for RS447 and no significant kurtosis was observed for FLJ40296 and RNU2. Most distribution patterns were flattened in the African population, since (excess) kurtosis was only found for RS447, D4Z4 (10q) and CT47. Homozygosity was observed in 25% of the unrelated female individuals for CT47, which was not surprising because of the little variation in repeat array length. The cumulative distribution functions (see material and methods) between the total sample and the three subpopulations differed significantly for six of the eight MSRs (Table 2). They were not significantly different for DXZ4 (p = 0.157) and RNU2 (p = 0.448). As a consequence, we considered the total population as an inhomogeneous group and modeled the distributions of the three subpopulations separately. The distribution of the whole population was, where relevant, estimated as a weighted average of the subsamples’ distributions. This difference between the subpopulations was confirmed by an independent (robust) *t*-test. Both RNU2 and DXZ4, which showed similar distributions in all three populations, had similar MSR means as well. The means of the remaining MSRs were significantly different for at least two populations (Figure 3, Additional file 2: Table S3). However, when the heterogeneity of MSRs between populations was significant, no MSR showed consistent behavior, where e.g. lengths were always shorter in a certain population compared to another population. For CT47 the mean array size in the Africans was significantly smaller than in the Caucasian individuals. But in general, we observed a subtle shift in repeat length towards longer repeats in the African population when compared to the Caucasian and Asian populations.

**Table 2:** Results of heterogeneity test for cumulative distribution function (CDF)

	**Mean squared difference**	**p-value**
RS447	0.0023	**0.038**^**a**^
MSR5p	0.0283	**0.000**
FLJ40296	0.0106	**0.000**
RNU2	0.0008	0.448
DXZ4	0.0017	0.157
CT47	0.0073	**0.000**
D4Z4 (4q)	0.0114	**0.000**
D4Z4 (10q)	0.0063	**0.000**

^a^value in bold: p < 0.05.

### Mitotic instability

Mitotic instability has been observed for D4Z4 [19], DXZ4 [6] and RS447 [13]. For D4Z4 it was suggested that this mainly occurs during the first few cycles of cell divisions after conception and that it results in gonosomal mosaicism [20]. We identified one Chinese male and one Caucasian male child showing mosaicism for MSR5p and RS447 respectively (1/270 = 0.4%), indicating mitotic instability (Additional file 2: Table S2). FLJ40296 was observed as a mosaic repeat in both an African father and mother (0.7%). Mosaicism for CT47 was observed in two Chinese females (0.7%), for DXZ4 in one Caucasian mother, one Caucasian father, two Chinese females, one African female child and one African male child (2.2%), and for RNU2 in four different individuals (1.5%): a Chinese female, a Japanese female, an African male child and an African father. Finally, for D4Z4 (4q) we observed a mosaic repeat in a Chinese female and an African father (0.7%) and D4Z4 (10q) was found as a mosaic repeat in a Chinese female and two African male children (1.1%).

### Meiotic instability

Direct analysis of meiotic instability was only possible in father-mother-child-trios, allowing repeat size comparison in children and parents in 60 trios. Of all 60 trios analyzed, one African male child was found with a different repeat length than one of the parents for the FLJ40296 repeat (1/120 = 0.8%) (Additional file 2: Table S2). From the maternally inherited repeat array, either a contraction of two repeat units or an expansion of four repeat units was observed. Also for RNU2 meiotic instability was observed in an African trio with the child presenting with a maternally inherited expansion of one repeat unit.

### Complex repeats

Some repeats showed more than two bands on the Southern blots, which could be explained by chromosomal instability of the HapMap cell lines, somatic mosaicism or a complex repeat structure. We defined a repeat as complex when the three (or sometimes even four) bands on the blot all had the same signal intensity and of which at least two were co-segregating when inherited by the offspring, through which the possibility of mosaicism or culture-induced instability was excluded. Complex repeats were found for MSR5p, FLJ40296, RNU2, DXZ4 and especially for RS447 in the African population, where up to 44% of the unrelated individuals showed a complex repeat.

### Multimodality

The majority of the MSRs showed that certain array lengths were more prevalent than other array lengths in between, suggesting that their distributions were composed of different modes. Based on the genetic heterogeneity analysis, the total population could be considered as an inhomogeneous group where size distributions differ significantly between the three subpopulations for six of the eight MSRs. Therefore the distributions of the three subpopulations were modeled separately. A weighted average of these three distributions was used to estimate the distribution for the aggregate population.

**Figure 4:**
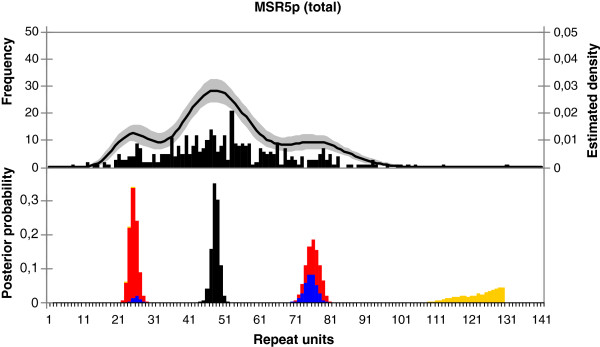
**MSR array size distribution of MSR5p in the combined (total) population.** Top panel: The histogram shows the size frequencies of the MSR5p arrays observed in the combined (total) population. Based on this frequency distribution of the observed data sample the corresponding estimated density of the unknown underlying distribution was calculated, which is displayed on top of the size histogram as a black line (right y-axis). The 95% posterior interval (i.e., the Bayesian counterpart of the 95% confidence interval) of this estimated density is indicated by the grey area. Bottom panel: Based on the estimated density of the unknown underlying distribution (and the uncertainty on this estimated density) the presence of modes and their locations was investigated. The graph shows the posterior probability of a mode for each point in the range of the data. The colors indicate the weight of the mode (i.e., the percentage of probability around the mode between the two surrounding minima) in the following order: weight ≥ 50% (black), 20% ≤ weight < 50% (blue), 10% ≤ weight < 20% (red), weight < 10% (yellow). For MSR5p, the main mode (black) is located around 50 units, two smaller modes (blue/red) around 25 and 75 units and a minor mode (yellow) around 130 units.

The modes below the 5% percentile and above the 95% percentile were excluded in the multimodality analysis to avoid influence of minor or spurious modes in the tails that may be based on one or two observations. Therefore the estimated posterior probabilities of the number of modes applied for the middle 90% range of the data. These estimated probability functions and their 95% posterior intervals (i.e. Bayesian counterparts of the 95% confidence intervals) for each MSR were projected on the size distribution histograms, as shown in Figure 4 (top panel) for MSR5p in the total population. Additional figures for the other MSRs and populations are added in the supplementary data (Additional file 3: Figures S1-S8). For CT47 the probability for a unimodal distribution was 100% in all the subpopulations and the aggregate population (Additional file 2: Table S4). However, strong evidence for the presence of multiple modes with a posterior probability > 99.33% was found for five of the other seven MSRs. The number of modes per MSR and the distance between them varied per population with a maximum of four different modes for D4Z4 (4q). Thus, our mixture model was indeed flexible, as it allowed for distributions with one to three or four modes since the Bayesian Information Criterion implied three or four (shifted) Poisson distributions for six of the eight MSRs.

In Figure 4 (bottom panel) we illustrate the distribution of the modes of MSR5p in the total population, where colors indicate the weight of the mode. So, for MSR5p in the total population, the main mode was located around 50 units, the two smaller modes around 25 and 75 units, and a minor one around 130 units. Additional figures for all populations and all MSRs can be found in the supplementary data (Additional file 3: Figures S1-S8).

The modes for MSR5p in the total population are also described in Table 3, including their corresponding size intervals. The probability that a combination of two modes is indeed existent is also indicated. To investigate whether the intervals between the modes are equidistant, the ratios of the modes’ locations were calculated, combined with their corresponding 95% and 98⅓% posterior intervals. So, for MSR5p in the total population, we could not reject that the ratios of second versus first, third versus first, and third versus second mode are 2, 3 and 1.5, respectively. In other words, the three modes that were present with a posterior probability of more than 85% were indeed likely to be equidistant, e.g. 25 units, 50 units and 75 units (Table 3). In the supplementary data the results for all MSRs are added (Additional file 2: Table S5). CT47 is omitted since no multimodality was present; DXZ4 is also omitted because it was unclear where the modes are located, e.g. if a certain number of units was a high value of the second mode or a low value of the third mode. However, for the other five MSRs showing a multimodal distribution pattern, similar results as for MSR5p also favored equidistance, especially for RNU2 and D4Z4 (10q). For the latter, in e.g. the Caucasian population we could not reject that the ratios of second versus first, third versus first, and third versus second mode are 2, 4 and 2, respectively, possibly reflecting modes at e.g. 10 units, 20 units and 40 units. These modes form a subset of an equidistant range of potential modes at 10, 20, 30 and 40 units. It may occur that one of the multiples does not show up as an actual mode, which may be a reason why the results for some of the other MSRs provide a less straightforward answer regarding the potential presence of equidistant modes.

**Table 3:** Posterior distribution of ratio of different modes’ locations for MSR5p (if existing)

						**Posterior of ratio of modes locations**			
	**Mode**	**Interval**^**a**^	**Mode**	**Interval**^**a**^	**Probability**^**b**^	**Mean**	**Median**	**95% p.i.**^**c**^	**98⅓% p.i.**
**ASN**	2	[40,48]	1	[21,29]	1.00	1.80	1.80	[1.65,1.96]	[1.63,2.00]
	3	[60,86]	1	[21,29]	0.97	2.92	2.92	[2.60,3.26]	[2.54,3.35]
	3	[60,86]	2	[40,48]	0.97	1.62	1.62	[1.50,1.75]	[1.47,1.80]
**CEU**	2	[48,59]	1	[9,40]	0.77	2.24	2.13	[1.56,3.47]	[1.47,3.92]
	3	[65,86]	1	[9,40]	0.53	3.25	3.12	[2.21,5.00]	[2.09,5.57]
	3	[65,86]	2	[48,59]	0.68	1.45	1.45	[1.36,1.55]	[1.34,1.57]
**YRI**	2	[46,65]	1	[19,45]	0.88	1.67	1.66	[1.44,1.96]	[1.40,2.08]
	3	[72,92]	1	[19,45]	0.88	2.49	2.47	[2.08,3.00]	[2.00,3.20]
	3	[72,92]	2	[46,65]	1.00	1.49	1.49	[1.38,1.60]	[1.35,1.62]
**Total**	2	[41,59]	1	[21,30]	0.93	1.91	1.92	[1.74,2.04]	[1.70,2.08]
	3	[68,84]	1	[21,30]	0.85	3.01	3.00	[2.71,3.25]	[2.67,3.29]
	3	[68,84]	2	[41,59]	0.91	1.57	1.57	[1.49,1.67]	[1.46,1.70]

^a^interval of repeat units where the corresponding mode should be located, which directly results from the data and is visualized in the bottom panels of Figure 4. These are the intervals with posterior probability of a (non-minor) mode, whereas between these intervals the posterior probability of a mode is negligible. Our findings on the modes’ locations therefore are derived from the data, not from a personal choice of intervals.

^b^posterior probability that both modes in the two given intervals exist.

^c^posterior interval (i.e. Bayesian counterpart of confidence interval).

## Discussion

In this study we examined the repeat array size and distribution of eight MSRs in three HapMap populations, a widely used panel to identify and catalog genetic similarities and differences in human beings. Until now, MSRs have been considerably understudied in the HapMap panel because, despite the rapid development of advanced genome research technologies, technical and bioinformatic limitations have thus far precluded detailed analysis of the sequence and composition of MSRs. MSR array size was investigated and analyzed in 210 unrelated individuals for the autosomal repeats RS447, MSR5p, FLJ40296, RNU2, D4Z4 (4q) and D4Z4 (10q) and the X chromosomal repeats DXZ4 and CT47.

The MSRs studied are highly polymorphic, some of them showing size variations of hundreds of kilobases (e.g. MSR5p) while for others the variation was much more limited (e.g. CT47). For DXZ4 and MSR5p a normal distribution is not rejected in the African and Caucasian population, respectively, while the other MSRs are significantly skewed and/or have significant (excess) kurtosis. MSRs do not show consistent behavior within or between populations, but overall we observe among Asians the least genetic variation, while the Africans show the highest genetic variation. This is consistent with the African origin of modern humans, where most human genetic diversity exists in the Africans, indicated previously by studies of microsatellites [21] and SNPs [22].

Repeat array instability can either result in contractions or expansions of the array over time. However, none of the MSRs showed a skewed distribution to the shorter repeat sizes while skewness to the longer repeat sizes was often observed, indicating that repeat expansion is more common than repeat contraction. A minimum of two repeat units is required to form an array, but for most of the studied MSRs we observe a higher minimum unit number, e.g. the shortest array detected for RS447 and MSR5p is eight units. This is reminiscent of observations from repeat structures composed of much smaller unit sizes that every repeat only becomes variable in length above a certain minimum threshold [23], despite the different mechanisms by which changes in copy number are being generated in these structures. Microsatellite copy number variation is mainly created by replication slippage of the DNA polymerase [24], while previous studies showed that the preferential mechanism by which D4Z4 MSR contracts or expands is by sister chromatid exchange [20]. The lack of MSR arrays below a certain threshold might either suggest that these sizes are less favorable by this rearrangement mechanism or that they might create an unfavorable chromatin structure and related transcriptional activity, perhaps associated with disease, as is seen for D4Z4 in the context of FSHD [4]. Furthermore it is possible that individuals with shorter repeat arrays were missed in this analysis because of the limited sample size. However, for D4Z4 (10q) it is known that approximately 16% of Caucasian individuals contain repeat arrays <11 units [25]. In this study, 17% of the Caucasian individuals and 16% of all individuals showed D4Z4 (10q) array sizes of ≤10 units, suggesting that for D4Z4 (10q) the HapMap panels are representative of a larger population. Therefore it is unlikely that shorter arrays were missed because of a limited sample size. Moreover, D4Z4 (10q) arrays <11 units were observed in 20% of the Asian and 9% of the African individuals, indicating a shift in the prevalence of repeat array sizes where longer repeat sizes are least prevalent in Asians, more in Caucasians and most in Africans.

We did not observe the meiotic mutation rate of 8.3% that was found previously for DXZ4 [6] and RS447 [13], probably because we only studied a limited number of meioses. However, a striking observation is the high mitotic instability found in all eight MSRs. A mitotic recombination rate of approximately 3% has been reported earlier for the D4Z4 (4q) and D4Z4 (10q) repeat arrays in the European population, where 1% of the individuals was mosaic for D4Z4 (4q) and 1.5% for D4Z4 (10q) [26]. Our findings indicate a comparable mitotic recombination rate of 0.4-2.2% in the other MSRs, which is four to ten times higher than the recombination rate of 0.1-0.2% most often described for microsatellite repeats [27,28]. This difference in recombination rate is probably explained by the different mechanisms that generate copy number variation in these structures [20,24]. Thus, MSRs are prone to frequent (mitotic) rearrangements and their polymorphic nature is likely a reflection of these high recombination rates.

Besides meiotic and mitotic instability, for five of the eight MSRs we also observed individuals showing additional bands on the Southern blots that could not be explained by mosaicism or culture-induced instability. These complex repeats suggest the presence of an additional restriction site within the repeat array for the enzyme used to digest the flanking regions or the presence of a homologous or duplicated repeat array elsewhere in the genome but probably on the same locus because the two fragments were always co-segregating when inherited by the offspring. Rearrangements between homologous chromosomes can also occur, which is described for D4Z4 (4q) and D4Z4 (10q) where exchanges between the repeat arrays occurred during evolution, leading to the formation of hybrid alleles, consisting of a combination of units derived from chromosome 4 and 10 [18].

Size variation in MSRs has been implicated in epigenetic control of the human genome affecting the expression of transcripts within and adjacent to the MSR. Therefore, their size regulation should be under strict control to avoid detrimental epigenomic consequences of copy number variation. Although MSRs often undergo rearrangements, our multimodality analysis indicates that these rearrangements indeed do not occur randomly. Previously we proposed that the D4Z4 array size distribution shows multimodality with three equidistant peaks at intervals of ~65 kb [26]. Given the preferred mechanism of rearrangement, this multimodality can be based on a founder effect, where two ancestral alleles or different chromosomal backgrounds (as has been shown for D4Z4 (4q) and D4Z4 (10q)) give rise to size variation over time and show little inter-chromosomal interactions [18]. Since MSRs are very polymorphic, it is more likely that this multimodality is based on other factors such as chromatin restrictions where certain chromatin states are more favorable than others. In this study we observed evidence for multimodality for seven of the eight MSRs, with CT47 being the exception showing a unimodal distribution. This presence of unimodality can be due to the small size range observed for CT47 where further repeat expansion can give rise to an additional mode. Multimodality in the MSR sizes can also arise by the presence of so-called recombination hotpots within the MSR array [29]. As was found for segmental duplications, also MSRs may be unstable DNA structures and therefore contain certain sequences that can function as a hotspot of structural genomic rearrangements.

For the five MSRs showing very strong evidence for multimodality, it is for MSR5p, RNU2 and D4Z4 (10q) also likely that this multimodality shows equidistant intervals between the modes. The location of the modes and the distance of the intervals between the modes vary depending on the populations and the MSR. The unequal location of the modes can still indicate the remnants of a founder effect, where the newly rearranged alleles originate from the ancestral ones and form a distribution around the size of the ancestral allele. During this process, arrays with energetically more favorable sizes are more frequently produced, since a higher order chromatin structure is imposed upon the MSR limiting its variation in array length. Thus, it seems that each MSR is organized in its own specific way, constrained by its own minimal array size. However, seven MSRs show multimodality and at least three of those also show equidistant intervals between the modes, suggesting a more universal organization of MSRs in our genome where they are arranged into higher order chromatin structures.

By applying Bayesian statistical methods in our study, we were able to perform a powerful analysis concerning multimodality in the MSR size distributions. Since the Bayesian analysis of flexible mixtures of (shifted) Poisson distributions allowed us to estimate the posterior probability without the necessity of making assumptions beforehand, it is a promising method to implement into future studies on frequencies of CNVs or gene expression.

## Conclusions

In this study we found substantial evidence of heterogeneity, multimodality and equidistant modes in the MSR array size distributions that were investigated. Considering that MSRs are large repeat arrays, highly dynamic and polymorphic and covering large regions of the genome, it is imperative to get a better understanding of their structure and function. Indeed, resolvement of the structural variation in the human genome, in particular this extreme form of copy number variation, can provide valuable insight into the mechanism of human genomic diseases.

## Methods

### DNA samples

For studying MSR length variation we used 270 human DNA samples from four populations of the International HapMap project: Utah residents with ancestry from Northern and Western Europe (CEU), Han Chinese individuals from Beijing, China (CHB), Japanese individuals from Tokyo, Japan (JPT) and Yoruba individuals from Ibadan, Nigeria (YRI) [30]. DNA was isolated from lymphoblastoid cell lines, obtained from the NIGMS Repository at the Coriell Institute for Medical Research (Camden, NJ, USA) [31]. For analyses, we considered the Chinese and Japanese individuals as one Asian population (ASN). This collection consists of 45 unrelated Chinese and 45 unrelated Japanese individuals, while Caucasian and African populations consist of 30 father-mother-child trios. Except for investigating mitotic and meiotic instability, the children of each trio were removed from the data set.

### Repeat array sizing

DNA sequences of the MSRs in the human genome were downloaded from the UCSC genome browser (Hg18 assembly) [32] and annotations were taken from GenBank [33]. The GenBank accession numbers of the macrosatellite repeat sequences are D38378 (RS447), AC106774.2 and AC113415.3 (MSR5p), AL353652.17 (FLJ40296), AC087365.3 (RNU2), S60754.1 (DXZ4), AL670379.17 (CT47), U85056.1 (D4Z4 (4q)) and AL732375.18 (D4Z4 (10q)). Primers were designed using RepeatMasker [34] and Primer3 [35] and ordered from Biolegio (Nijmegen, the Netherlands) (Additional file 2: Table S1). The probes were checked for specificity with NCBI Blast [36]. To assure high quality DNA, cells were embedded in agarose, treated with sarkosyl (1%) and pronase (20 mg/ml) and stored as plugs in 0.5 M EDTA at 4°C. Before use, plugs were equilibrated in TE^-4^ and the appropriate restriction enzyme buffer. For RNU2, DXZ4 and CT47 DNA samples were digested with *Eco*RI, while *Hin*dIII was used for MSR5p and FLJ40296 arrays and *Bam*HI for RS447. Digestion and hybridization for D4Z4 array sizing was performed as described previously [37]. DNA fragments were separated by pulsed-field gel electrophoresis (PFGE) on a 0.8% agarose gel. After digestion and gel electrophoresis, DNA was transferred to a Hybond XL membrane (GE Healthcare) by Southern blotting. The DNA was cross linked to the membrane by the Stratagene UV Stratalinker 1200 using autocrosslink setting. The membrane was hybridized with the ^32^P labeled probes in a buffer containing 0.125 M Na_2_HPO_4_ (pH 7.2), 10% PEG6000, 0.25 M NaCl, 1 mM EDTA, and 7% SDS for 16-24 h at 65°C. Washing conditions are described in Additional file 2: Table S1. All membranes were exposed for 48 hours to phosphor-imager screens and analyzed with Image Quant software (Molecular Dynamics).

### Statistical analyses

The estimated repeat array sizes in kb obtained by PFGE were converted to the total number of repeat units in the array based on the DNA sequence from the UCSC genome browser. Normality tests, skewness and kurtosis were calculated using NCSS97. StatXact 4 (Cytel Software) was used to determine gender differences by Wilcoxon Mann-Whitney exact test with Monte Carlo simulation (10,000 randomizations). The chromosomes of individuals containing gonosomal rearranged repeat arrays were removed from the sizing analysis.

### Genetic heterogeneity

In order to analyze whether the observations from the three populations stem from the same probability distribution, we investigated whether there are significant differences between the empirical distributions for each population for every MSR separately. For this, a standard test (using the Cramér-von Mises statistic) was applied to determine whether the empirical cumulative distribution function (CDF) differs significantly between the three populations and the aggregate population [38,39]. We also tested for significant differences between the sample means, using a bootstrap based robust *t*-test that is also reliable in case of non-normally distributed data.

### Multimodality

To conclude with reasonably large credibility whether multimodality was present in the probability distributions of the MSR size data, a discrete distribution was used to model the data. Given the typical pattern of the MSR size data, we needed a flexible distribution that could *a priori* take several shapes, so that it could approximate many types of empirical distributions with high accuracy. As a flexible class a mixture of (shifted) Poisson distributions was considered. The flexibility of mixtures (convex combinations of probability distributions) is well illustrated in literature [40,41]. We used a Bayesian approach for the estimation, as this made it possible to estimate the posterior probability of multimodality, whereas the frequentist test of Silverman was only able to reject or not-reject the null hypothesis of unimodality [42] and this test has typically weak power [43]. Further, our Bayesian approach allowed us to obtain not only point estimates of the modes’ locations, but the whole posterior distribution that informed us also on the uncertainty on the estimated modes’ locations (for full details on model, estimation and results we refer to LFH, NB, and HKvD, manuscript in preparation).

In each model the Bayesian Information Criterion (BIC) was used to determine the number of (shifted) Poisson distributions. Note that the number of (shifted) Poisson distributions is typically *not* equal to the number of modes. For example, a combination of two relatively nearby distributions can form a distribution with one mode (and possibly higher skewness or kurtosis). On the other hand, the number of modes cannot exceed the number of (shifted) Poisson distributions. This model was estimated by Gibbs sampling with data augmentation [44,45], simulating a series of 10,000 draws (after a discarded burn-in of 1,000 draws to eliminate the effect of initial values) from the conditional posterior distributions of the parameters and latent variables indicating to which of the (shifted) Poisson distributions each observation belonged.

We also investigated whether multiple modes are equidistant in the sense that two modes are *m* and 2*m* (or that three consecutive modes are *m*, 2*m* and 3*m)* for a certain value of *m*. For this the posterior distribution of ratios of the different modes was considered. Not only 95% but also 98⅓% posterior intervals were calculated to facilitate a more conservative joint test of three equations with total significance level no larger than 1⅔% * 3 = 5%.

A significance level of 0.05 was considered for all tests.

### RNU2 locus

The sequence of the MSR RNU2 was based on a single unfinished BAC clone (AC087365.3) in the NCBI36/hg18 assembly. While executing the experiments, this BAC clone was removed from the new GRCh37/hg19 assembly. It is known that RNU2 must be located on chromosome 17q21-22 [46], but the exact location of RNU2 is unknown. As a result in recalculations we could not reduce the measured size of the bands on the Southern blots with the non homologous flanking regions within the *Eco*RI restriction sites. Consequently, absolute MSR sizes for this locus should be read with caution, but relative sizes can still be used reliably.

## Abbreviations

MSR: Macrosatellite repeat; STR: Short tandem repeat; bp: Base pairs; kb: Kilobases; FSHD: Facioscapulohumeral dystrophy; CEU: Utah residents with ancestry from Northern and Western Europe; CHB: Han Chinese individuals from Beijing, China; JPT: Japanese individuals from Tokyo, Japan; ASN: Chinese and Japanese individuals; YRI: Yoruba individuals from Ibadan, Nigeria; PFGE: Pulsed-Field Gel Electrophoresis; CDF: Cumulative distribution function; BIC: Bayesian Information Criterion

## Competing interests

The authors declare that they have no competing interests.

## Authors’ contributions

RJLFL, PdK and SMvdM conceived of and designed the study. PdK and SMvdM directed the study. MS, RJLFL, RM and PJvdV performed experiments. LFH, HKvD and NB provided statistical support. All authors analyzed and interpreted data. MS, PdK and SMvdM wrote the manuscript. All authors read and approved the final manuscript.

## Supplementary Material

Additional file 1 **Observed repeat sizes for all MSRs.** Additional file containing tables with repeat sizes obtained in this study for every individual for every MSR separately. The file is in .xls format. Click here for file

Additional file 2 **Additional file containing supplementary tables S1-S5 and their legends.** The file is in .pdf format. Click here for file

Additional file 3 **Additional file containing supplementary figures S1-S8 and their legends.** The file is in .pdf format. Click here for file
