# Supplementary material for: Genome-wide analysis of macrosatellite repeat copy number variation in worldwide populations: evidence for differences and commonalities in size distributions and size restrictions
Source: BMC Genomics. 2013 Mar 4;14:143. doi: 10.1186/1471-2164-14-143 (PMC3599962; doi:10.1186/1471-2164-14-143)
Supplement: Additional file 2 — Additional file containing supplementary tables S1-S5 and their legends. The file is in .pdf format. [file 1471-2164-14-143-S2.pdf]

Supplemental tables

Table S1. Primer sequences and characteristics of corresponding probe

**MSR**	**Primer sequences[a] (5' → 3')**	**Probe size (bp)**	**Location[b]**	**Washing conditions[c]**
RS447	F: ATCCAGGCAGCTCAGAGTGT<br>R: GCTCTTTCCACCAAGTGCTC	604	internal	2x 0.3xSSC, 0.1% SDS<br>1x 0.1xSSC, 0.1% SDS
MSR5p	F: CGATCTGCTGTCTTCATCCA<br>R: GGAAGGTGAGCTCAGGAGTG	644	distal	1x 0.3xSSC, 0.1% SDS<br>2x 0.1xSSC, 0.1% SDS
FLJ40296	F: TTTGGATGCTTTCCTTGACC<br>R: GCAGGCGTTTGATGTACCTT	749	internal	2x 2xSSC, 0.1% SDS<br>1x 1xSSC, 0.1% SDS
RNU2	F: TAAGGGCTAGGAAGGGGGTA<br>R: AATGCCAATGACAACGATGA	650	distal	3x 2xSSC, 0.1% SDS
DXZ4	F: ACTAGCCTGCCTTCCTGACA<br>R: CCAGTAGAAGTGGGCGAGAG	940	internal	1x 2xSSC, 0.1% SDS<br>2x 1xSSC, 0.1% SDS
CT47	F: CTGCTGCTTGATCATTTCCA<br>R: AGAGGGTAAGGAACGGGCTA	710	internal	1x 2xSSC, 0.1% SDS<br>2x 1xSSC, 0.1% SDS

[a]F = forward primer, R = reverse primer
[b]location = location of probe relative to MSR array
[c]SSC = saline-sodium citrate, SDS = sodium dodecyl sulfate

Table S2. Overview of the results of array sizes and stability of the studied MSRs

MSR	Size variation[a] (units)	Minimum[b]	Mitotic[c] (%)	Meiotic[d] (%)
RS447	8-113	8	0.4	-
MSR5p	8-131	8	0.4	-
FLJ40296	2-30	2	0.7	0.8
RNU2	5-63	5	1.5	0.8
DXZ4	18-120	12	2.2	-
CT47	4-17	4	0.7	-
D4Z4 (4q)	6-113	1	0.7	-
D4Z4 (10q)	2-105	1	1.1	-

[a]size variation observed in this study
[b]minimum = minimum number of repeat units observed in this study or described previously
[c]mitotic = mitotic recombination rate observed in this study
[d]meiotic = meiotic recombination rate observed in this study

Table S3. Results of heterogeneity test for mean repeat array size

	Sample mean			H_0: mean ASN - mean CEU=0		H_0: mean ASN - mean YRI=0		H_0: mean CEU - mean YRI=0	
	ASN	CEU	YRI	t-value	p-value	t-value	p-value	t-value	p-value
RS447	61.47	56.16	61.77	2.036	**0.035**[a]	-0.095	0.925	-1.716	0.089
MSR5p	41.29	57.06	61.79	-8.355	**0.000**	-9.667	**0.000**	-2.042	**0.041**
FLJ40296	7.97	9.41	11.75	-3.224	**0.001**	-6.234	**0.000**	-3.536	**0.000**
RNU2	22.79	22.99	21.72	-0.142	0.887	0.793	0.428	0.818	0.407
DXZ4	57.42	59.92	60.82	-1.120	0.261	-1.527	0.129	-0.382	0.700
CT47	6.63	7.48	6.79	-3.832	**0.000**	-0.643	0.522	2.877	**0.006**
D4Z4 (4q)	23.70	34.88	35.02	-6.096	**0.000**	-5.374	**0.000**	-0.053	0.954
D4Z4 (10q)	21.93	22.05	30.95	-0.081	0.936	-4.719	**0.000**	-4.441	**0.000**

[a]value in bold: $p < 0.05$

Table S4. Estimated posterior probabilities of number of modes

		Number of modes				
		**1**	**2**	**3**	**4**	**5**
**RS447**	**ASN**	0.028	0.345	0.463	0.165	0.000
	**CEU**	0.001	0.433	0.566	0.000	0.000
	**YRI**	0.000	0.010	0.990	0.000	0.000
	**Total**	0.001	0.078	0.909	0.012	0.000
**MSR5p**	**ASN**	0.001	0.896	0.104	0.000	0.000
	**CEU**	0.357	0.640	0.004	0.000	0.000
	**YRI**	0.000	0.821	0.179	0.000	0.000
	**Total**	0.007	0.145	0.846	0.001	0.000
**FLJ40296**	**ASN**	1.000	0.000	0.000	0.000	0.000
	**CEU**	0.913	0.087	0.000	0.000	0.000
	**YRI**	0.307	0.693	0.000	0.000	0.000
	**Total**	0.915	0.085	0.000	0.000	0.000
**RNU2**	**ASN**	0.000	0.999	0.001	0.000	0.000
	**CEU**	0.000	0.236	0.764	0.000	0.000
	**YRI**	0.001	0.247	0.752	0.000	0.000
	**Total**	0.073	0.897	0.030	0.000	0.000
**DXZ4**	**ASN**	0.229	0.713	0.058	0.001	0.000
	**CEU**	0.143	0.509	0.349	0.000	0.000
	**YRI**	0.069	0.906	0.026	0.000	0.000
	**Total**	0.553	0.432	0.014	0.001	0.000
**CT47**	**ASN**	1.000	0.000	0.000	0.000	0.000
	**CEU**	1.000	0.000	0.000	0.000	0.000
	**YRI**	1.000	0.000	0.000	0.000	0.000
	**Total**	1.000	0.000	0.000	0.000	0.000
**D4Z4 (4q)**	**ASN**	0.078	0.385	0.537	0.000	0.000
	**CEU**	0.000	1.000	0.000	0.000	0.000
	**YRI**	0.000	0.245	0.648	0.107	0.000
	**Total**	0.083	0.601	0.286	0.030	0.000
**D4Z4 (10q)**	**ASN**	0.008	0.230	0.762	0.000	0.000
	**CEU**	0.000	0.032	0.967	0.000	0.000
	**YRI**	0.000	0.001	1.000	0.000	0.000
	**Total**	0.006	0.462	0.508	0.024	0.000

Estimated posterior probabilities of number of modes on the middle 90% range of the repeat array sizes, e.g. for CT47 there is a 100% probability for all populations that the repeat array size distribution consists of only one mode. On the contrary, the posterior probability that only one mode is present for RS447 in the Asian population is 2.8%, for

two modes the probability is 34.5% and there is a 46.3% probability for the presence of three modes and 16.5% for the presence of four modes.

In Table S4 the 5% left and right tails of the distributions were discarded, since a conservative test only for the presence of multiple ‘important’ modes was desired. In Table 3 and Table S5 however, the full range of data was considered, since the focus was on the location of the modes if multimodality was present. Therefore, Table 3 and Table S5 may refer to third or fourth modes (in the whole range), even when Table S4 indicates the presence of at most two or three modes (in the middle 90% range).

Table S5. Posterior distribution of ratio of different modes' locations (if existing)

							posterior of ratio of modes' locations			
		**mode**	**interval**[a]	**mode**	**interval**[a]	**probability**[b]	**mean**	**median**	**95% p.i.**[c]	**98⅓% p.i.**
**RS447**	**ASN**	2	[39,59]	1	[22,31]	0.92	1.84	1.85	[1.64,2.08]	[1.59,2.13]
		3	[61,77]	1	[22,31]	1.00	2.58	2.58	[2.32,2.88]	[2.27,2.96]
		3	[61,77]	2	[39,59]	0.91	1.40	1.40	[1.28,1.52]	[1.25,1.55]
	**CEU**	2	[40,64]	1	[26,34]	0.58	1.68	1.68	[1.45,1.94]	[1.42,2.03]
		3	[65,78]	1	[26,34]	0.99	2.38	2.38	[2.19,2.55]	[2.16,2.59]
		3	[65,78]	2	[40,64]	0.57	1.43	1.43	[1.27,1.60]	[1.23,1.64]
	**YRI**	2	[39,60]	1	[19,32]	1.00	1.89	1.88	[1.67,2.13]	[1.61,2.19]
		3	[76,89]	1	[19,32]	1.00	3.16	3.15	[2.82,3.57]	[2.76,3.68]
		3	[76,89]	2	[39,60]	1.00	1.67	1.67	[1.54,1.82]	[1.51,1.86]
	**Total**	2	[43,58]	1	[25,31]	0.92	1.75	1.75	[1.59,1.90]	[1.55,1.96]
		3	[61,78]	1	[25,31]	0.98	2.50	2.50	[2.34,2.67]	[2.30,2.70]
		3	[61,78]	2	[43,58]	0.93	1.43	1.44	[1.31,1.55]	[1.29,1.58]
**FLJ40296**	**YRI**	2	[14,23]	1	[6,10]	0.69	2.25	2.25	[2.00,2.57]	[1.89,2.71]
**RNU2**	**ASN**	2	[23,31]	1	[11,16]	1.00	2.06	2.08	[1.86,2.25]	[1.79,2.33]
	**CEU**	2	[18,34]	1	[10,17]	0.93	2.06	2.08	[1.75,2.42]	[1.69,2.50]
		3	[35,57]	1	[10,17]	0.88	3.35	3.33	[2.86,3.91]	[2.79,4.08]
		3	[35,57]	2	[18,34]	0.81	1.66	1.65	[1.44,1.90]	[1.39,1.95]
	**YRI**	2	[16,30]	1	[7,15]	0.81	1.91	1.91	[1.58,2.25]	[1.50,2.33]
		3	[31,43]	1	[7,15]	0.94	3.3	3.27	[2.69,4.00]	[2.54,4.22]
		3	[31,43]	2	[16,30]	0.77	1.78	1.79	[1.48,2.12]	[1.42,2.18]
	**Total**	2	[19,34]	1	[10,16]	0.93	2.06	2.08	[1.83,2.33]	[1.77,2.36]
**D4Z4 (4q)**	**ASN**	2	[20,27]	1	[10,19]	0.63	1.60	1.60	[1.35,1.85]	[1.29,1.92]
		3	[30,45]	1	[10,19]	0.59	2.53	2.53	[2.13,3.00]	[2.00,3.15]
		3	[30,45]	2	[20,27]	0.80	1.63	1.62	[1.46,1.81]	[1.42,1.86]
	**CEU**	2	[47,58]	1	[21,27]	1.00	2.18	2.17	[2.04,2.33]	[2.00,2.36]
	**YRI**	2	[21,28]	1	[6,14]	1.00	2.66	2.67	[2.18,3.14]	[2.09,3.29]

		3	[37,48]	1	[6,14]	1.00	4.80	4.78	[3.91,5.75]	[3.73,6.14]
		3	[37,48]	2	[21,28]	1.00	1.80	1.79	[1.64,1.96]	[1.60,2.00]
		4	[69,87]	1	[6,14]	1.00	8.65	8.56	[7.00,10.50]	[6.67,11.14]
		4	[69,87]	2	[21,28]	1.00	3.25	3.25	[2.92,3.59]	[2.88,3.68]
		4	[69,87]	3	[37,48]	1.00	1.80	1.80	[1.66,1.95]	[1.63,2.00]
**D4Z4 (10q)**	**ASN**	2	[15,27]	1	[8,13]	0.95	1.95	2.00	[1.64,2.22]	[1.55,2.33]
		3	[28,47]	1	[8,13]	0.79	3.39	3.36	[2.82,4.00]	[2.67,4.22]
		3	[28,47]	2	[15,27]	0.77	1.75	1.75	[1.57,1.95]	[1.52,2.05]
	**CEU**	2	[17,27]	1	[6,12]	1.00	2.45	2.50	[2.00,3.00]	[2.00,3.00]
		3	[30,47]	1	[6,12]	0.99	4.68	4.67	[3.70,5.86]	[3.50,6.00]
		3	[30,47]	2	[17,27]	0.99	1.91	1.90	[1.67,2.15]	[1.62,2.20]
	**YRI**	2	[22,32]	1	[8,17]	1.00	2.22	2.23	[1.92,2.60]	[1.80,2.70]
		3	[46,58]	1	[8,17]	1.00	4.31	4.25	[3.57,5.20]	[3.43,5.44]
		3	[46,58]	2	[22,32]	1.00	1.94	1.93	[1.76,2.12]	[1.72,2.16]
	**Total**	2	[16,28]	1	[8,14]	0.99	2.03	2.00	[1.73,2.33]	[1.62,2.44]

[a]interval of repeat units where the corresponding mode should be located, which directly results from the data and is visualized in the bottom panels of Figures S1-S8. These are the intervals with posterior probability of a (non-minor) mode, whereas between these intervals the posterior probability of a mode is negligible. Our findings on the modes' locations therefore are derived from the data, not from a personal choice of intervals.

[b]posterior probability that both modes in the two given intervals exist

[c]posterior interval (i.e. Bayesian counterpart of confidence interval)
