# Supplementary material for: Genome-wide analysis of macrosatellite repeat copy number variation in worldwide populations: evidence for differences and commonalities in size distributions and size restrictions
Source: BMC Genomics. 2013 Mar 4;14:143. doi: 10.1186/1471-2164-14-143 (PMC3599962; doi:10.1186/1471-2164-14-143)
Supplement: Additional file 3 — Additional file containing supplementary figures S1-S8 and their legends. The file is in .pdf format. [file 1471-2164-14-143-S3.pdf]

Supplemental figures

Figure S1. MSR array size distributions for MSR5p

Top panel: The histogram shows the size frequencies of the MSR5p arrays observed in the Asian, Caucasian and African population respectively. Based on this frequency distribution of the observed data sample the corresponding estimated density of the unknown underlying distribution was calculated, which is displayed on top of the size histogram as a black line (right y-axis). The 95% posterior interval (i.e., the Bayesian counterpart of the 95% confidence interval) of this estimated density is indicated by the grey area.

Bottom panel: Based on the estimated density of the unknown underlying distribution (and the uncertainty on this estimated density) the presence of modes and their locations was investigated. The graph shows the posterior probability of a mode for each point in the range of the data. The colors indicate the weight of the mode (i.e., the percentage of probability around the mode between the two surrounding minima) in the following order: weight ≥ 50% (black), 20% ≤ weight < 50% (blue), 10% ≤ weight < 20% (red), weight < 10% (yellow).

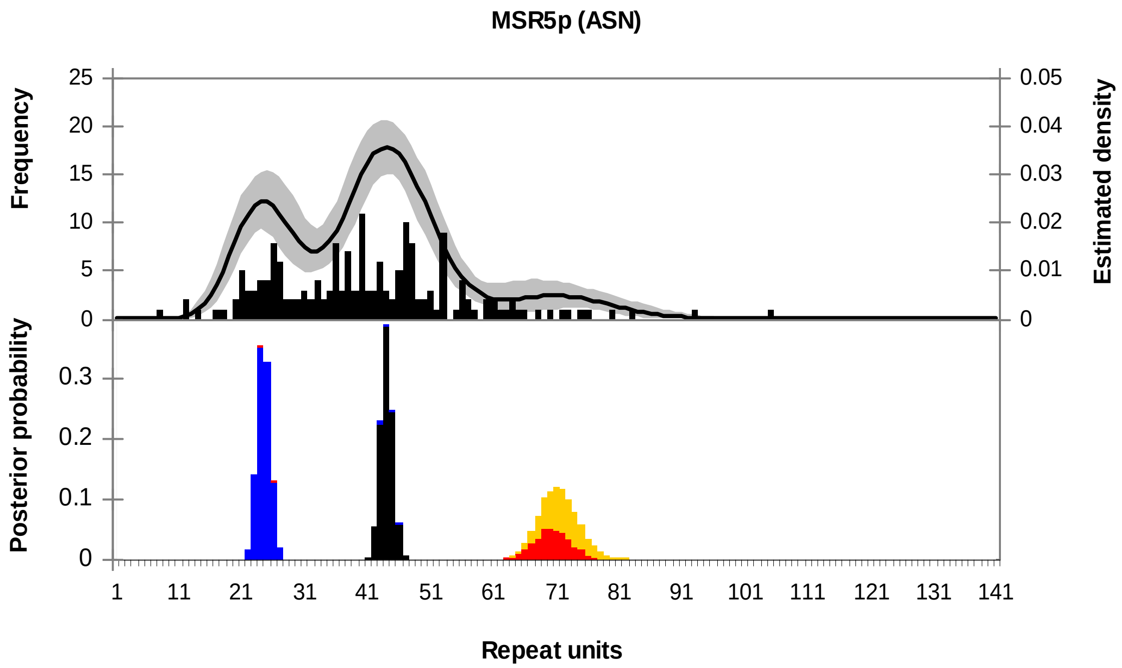

Figure S1a. MSR array size distribution for MSR5p in the Asian population

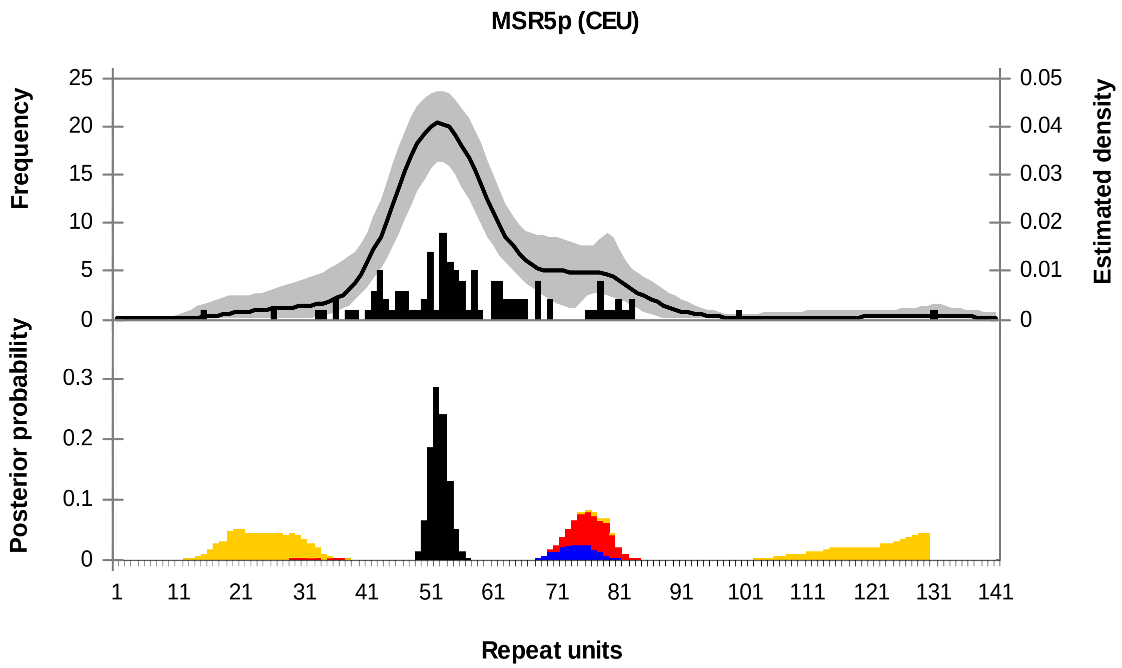

Figure S1b. MSR array size distribution for MSR5p in the Caucasian population

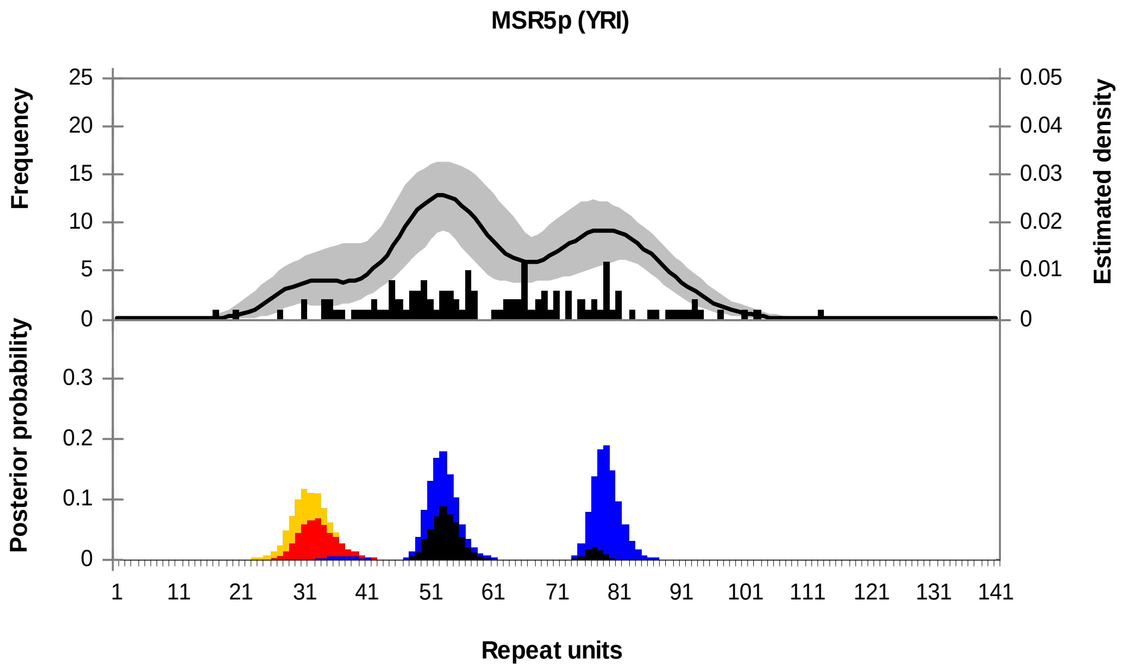

Figure S1c. MSR array size distribution for MSR5p in the African population

Figure S2. MSR array size distributions for RS447

Top panel: The histogram shows the size frequencies of the RS447 arrays observed in the Asian, Caucasian, African and total population respectively. Based on this frequency distribution of the observed data sample the corresponding estimated density of the unknown underlying distribution was calculated, which is displayed on top of the size histogram as a black line (right y-axis). The 95% posterior interval (i.e., the Bayesian counterpart of the 95% confidence interval) of this estimated density is indicated by the grey area.

Bottom panel: Based on the estimated density of the unknown underlying distribution (and the uncertainty on this estimated density) the presence of modes and their locations was investigated. The graph shows the posterior probability of a mode for each point in the range of the data. The colors indicate the weight of the mode (i.e., the percentage of probability around the mode between the two surrounding minima) in the following order: weight ≥ 50% (black), 20% ≤ weight < 50% (blue), 10% ≤ weight < 20% (red), weight < 10% (yellow).

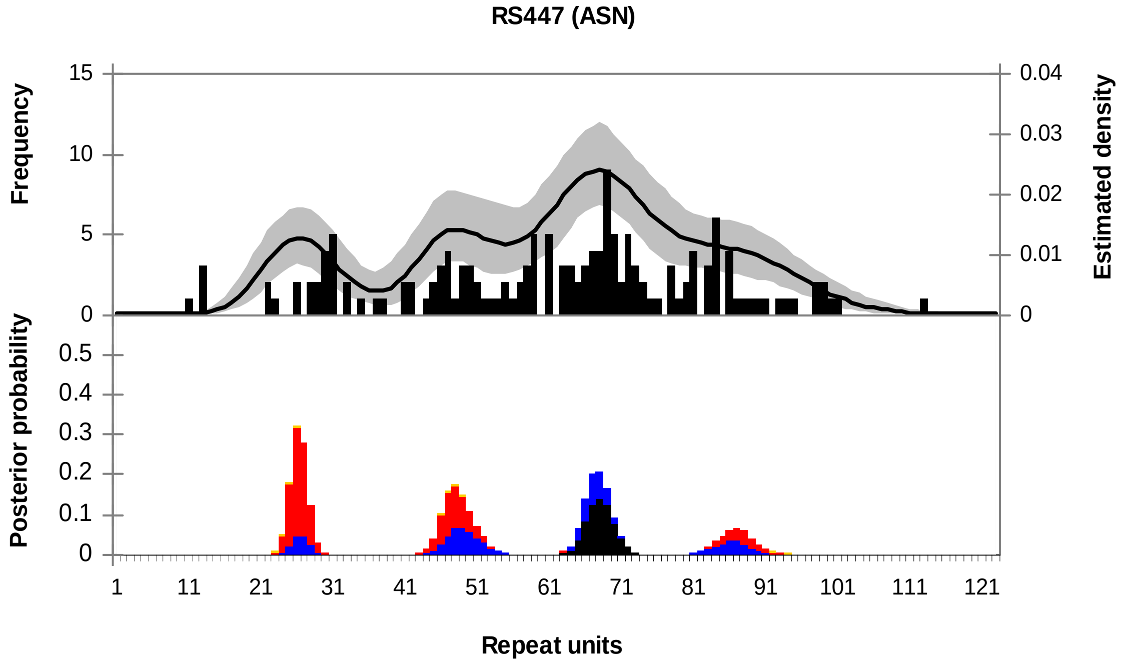

Figure S2a. MSR array size distribution for RS447 in the Asian population

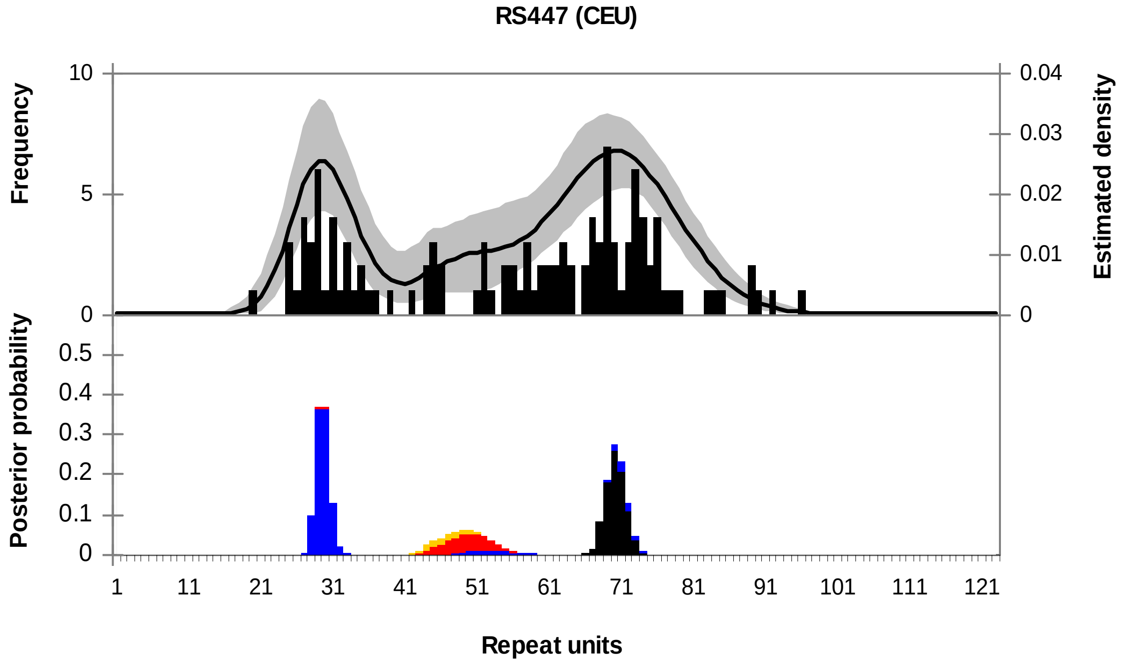

Figure S2b. MSR array size distribution for RS447 in the Caucasian population

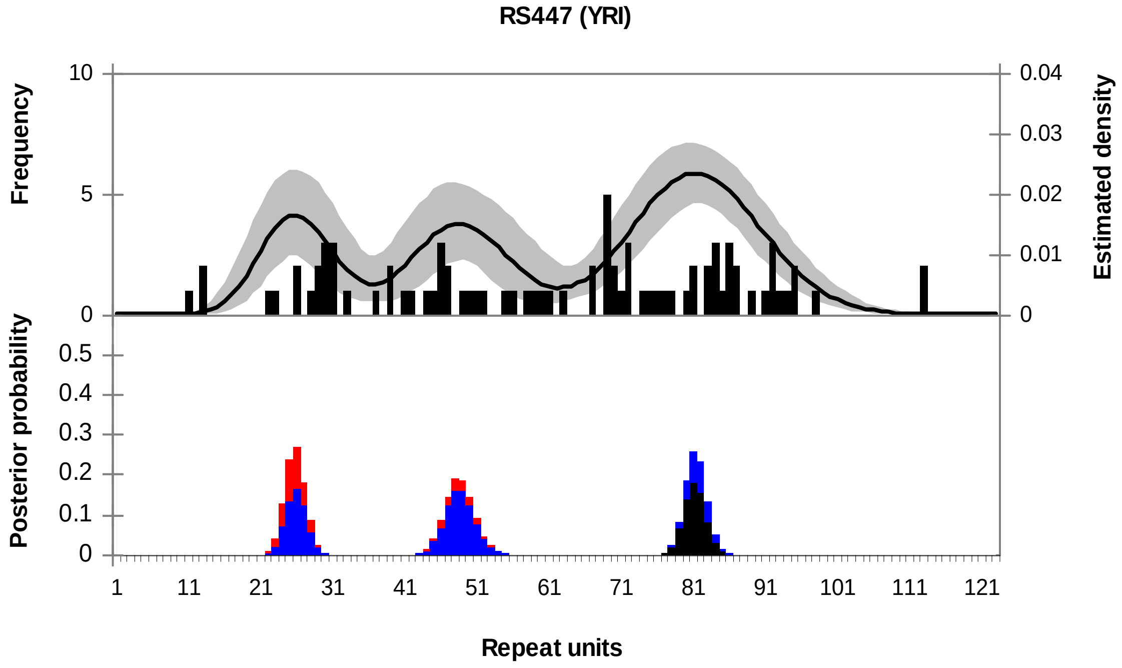

Figure S2c. MSR array size distribution for RS447 in the African population

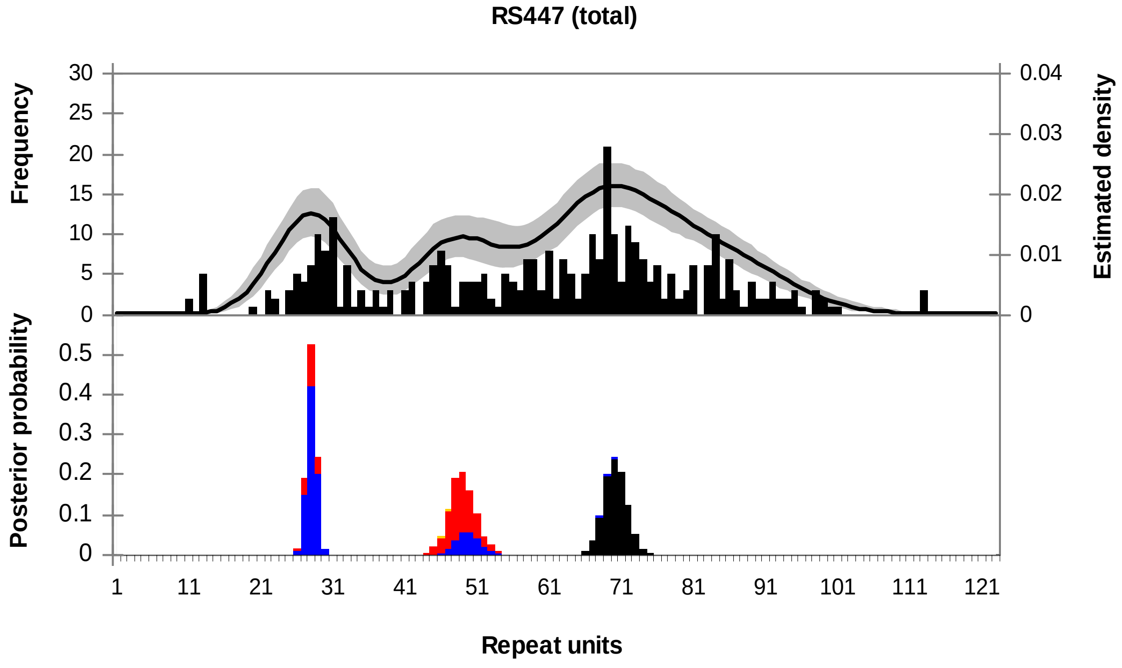

Figure S2d. MSR array size distribution for RS447 in the total population

Figure S3. MSR array size distributions for FLJ40296

Top panel: The histogram shows the size frequencies of the FLJ40296 arrays observed in the Asian, Caucasian, African and total population respectively. Based on this frequency distribution of the observed data sample the corresponding estimated density of the unknown underlying distribution was calculated, which is displayed on top of the size histogram as a black line (right y-axis). The 95% posterior interval (i.e., the Bayesian counterpart of the 95% confidence interval) of this estimated density is indicated by the grey area.

Bottom panel: Based on the estimated density of the unknown underlying distribution (and the uncertainty on this estimated density) the presence of modes and their locations was investigated. The graph shows the posterior probability of a mode for each point in the range of the data. The colors indicate the weight of the mode (i.e., the percentage of probability around the mode between the two surrounding minima) in the following order: weight ≥ 50% (black), 20% ≤ weight < 50% (blue), 10% ≤ weight < 20% (red), weight < 10% (yellow).

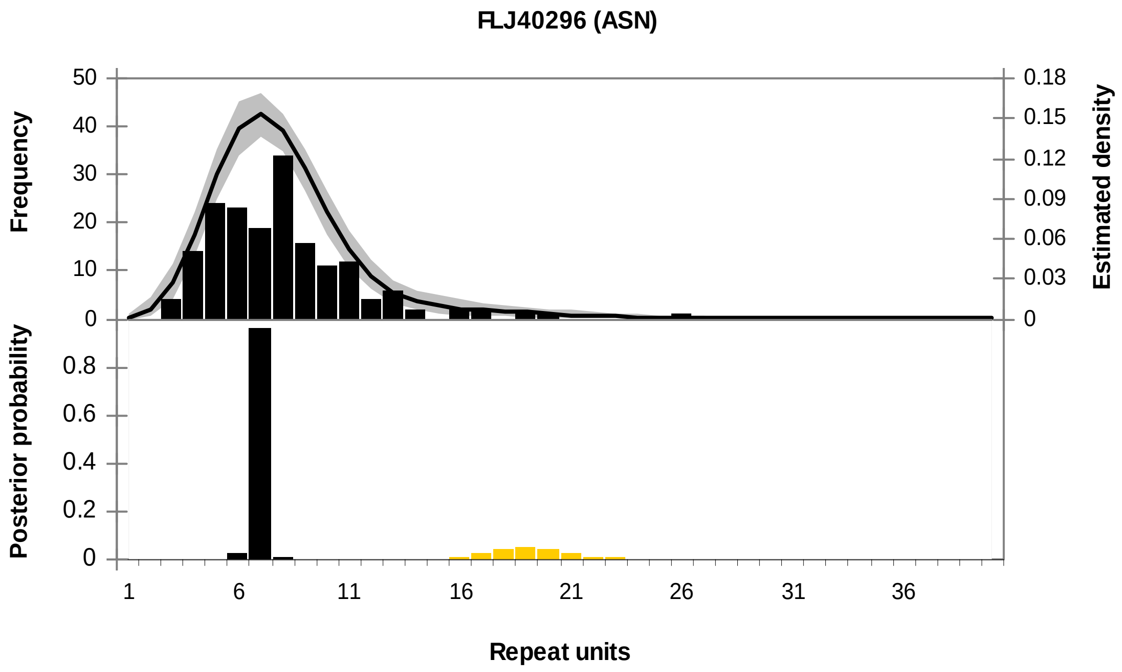

Figure S3a. MSR array size distribution for FLJ40296 in the Asian population

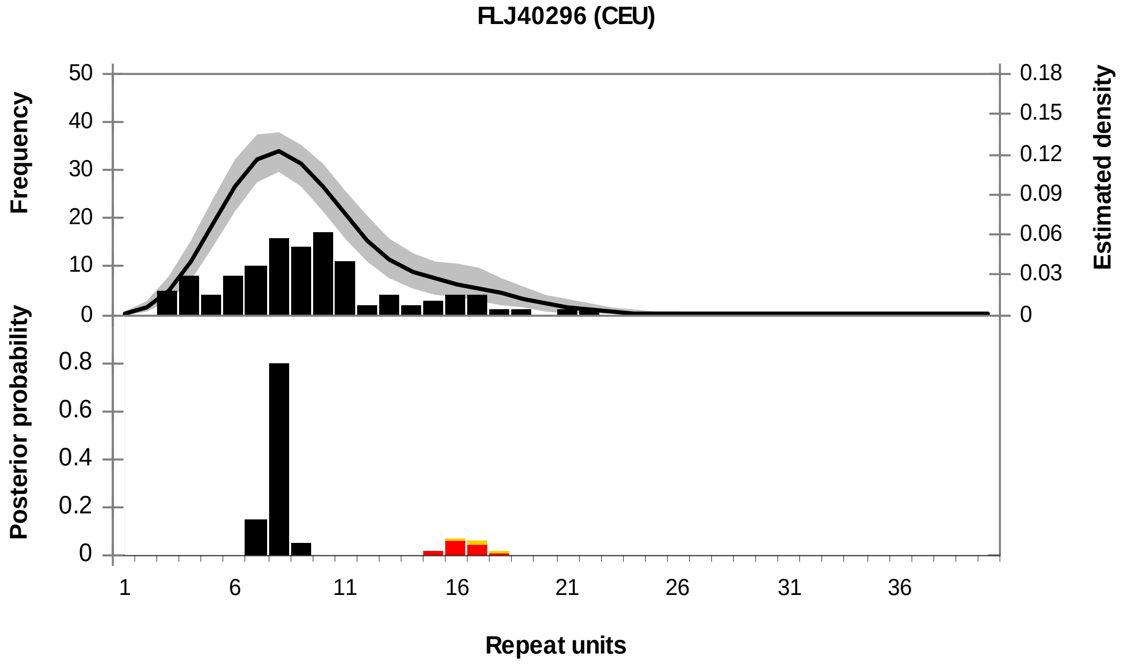

Figure S3b. MSR array size distribution for FLJ40296 in the Caucasian population

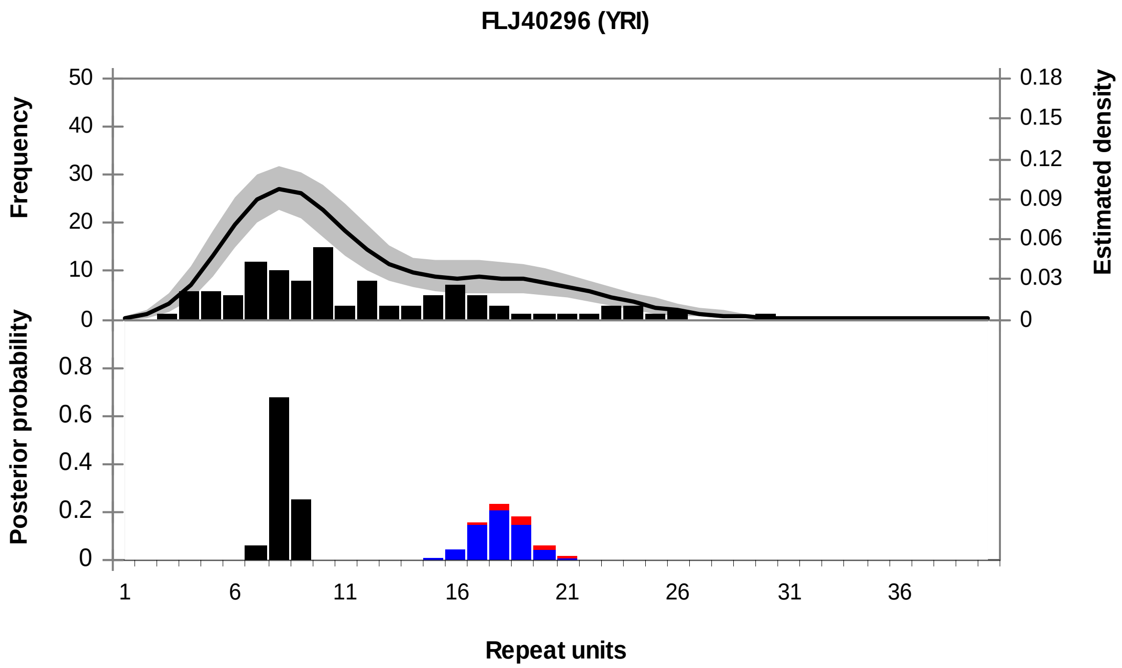

Figure S3c. MSR array size distribution for FLJ40296 in the Caucasian population

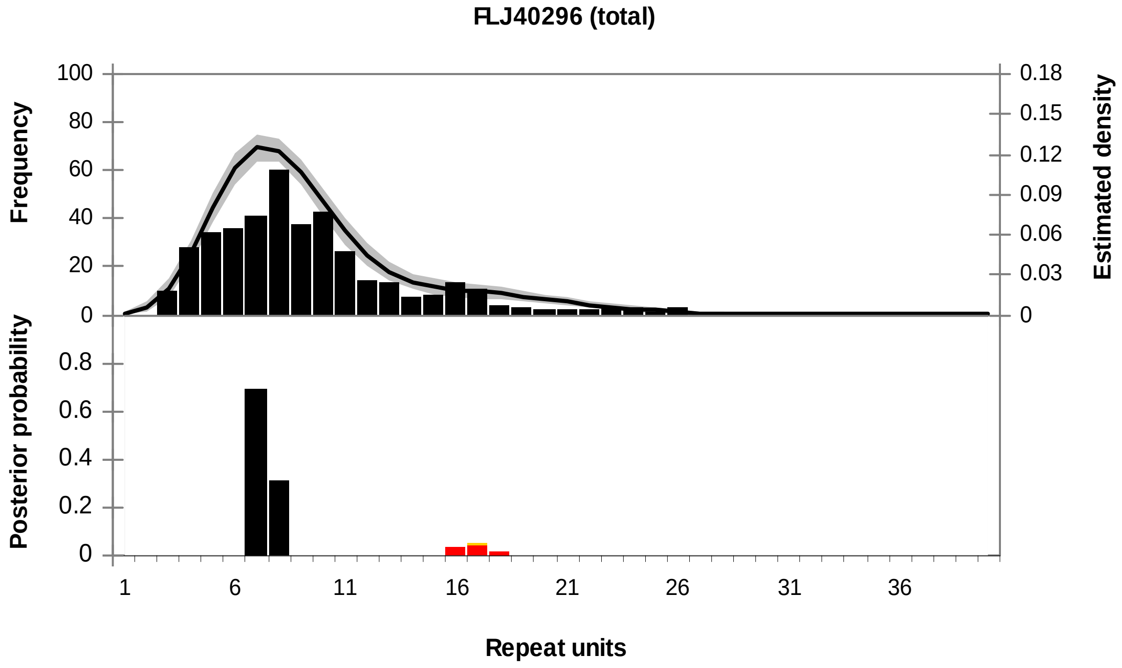

Figure S3d. MSR array size distribution for FLJ40296 in the total population

Figure S4. MSR array size distributions for RNU2

Top panel: The histogram shows the size frequencies of the RNU2 arrays observed in the Asian, Caucasian, African and total population respectively. Based on this frequency distribution of the observed data sample the corresponding estimated density of the unknown underlying distribution was calculated, which is displayed on top of the size histogram as a black line (right y-axis). The 95% posterior interval (i.e., the Bayesian counterpart of the 95% confidence interval) of this estimated density is indicated by the grey area.

Bottom panel: Based on the estimated density of the unknown underlying distribution (and the uncertainty on this estimated density) the presence of modes and their locations was investigated. The graph shows the posterior probability of a mode for each point in the range of the data. The colors indicate the weight of the mode (i.e., the percentage of probability around the mode between the two surrounding minima) in the following order: weight ≥ 50% (black), 20% ≤ weight < 50% (blue), 10% ≤ weight < 20% (red), weight < 10% (yellow).

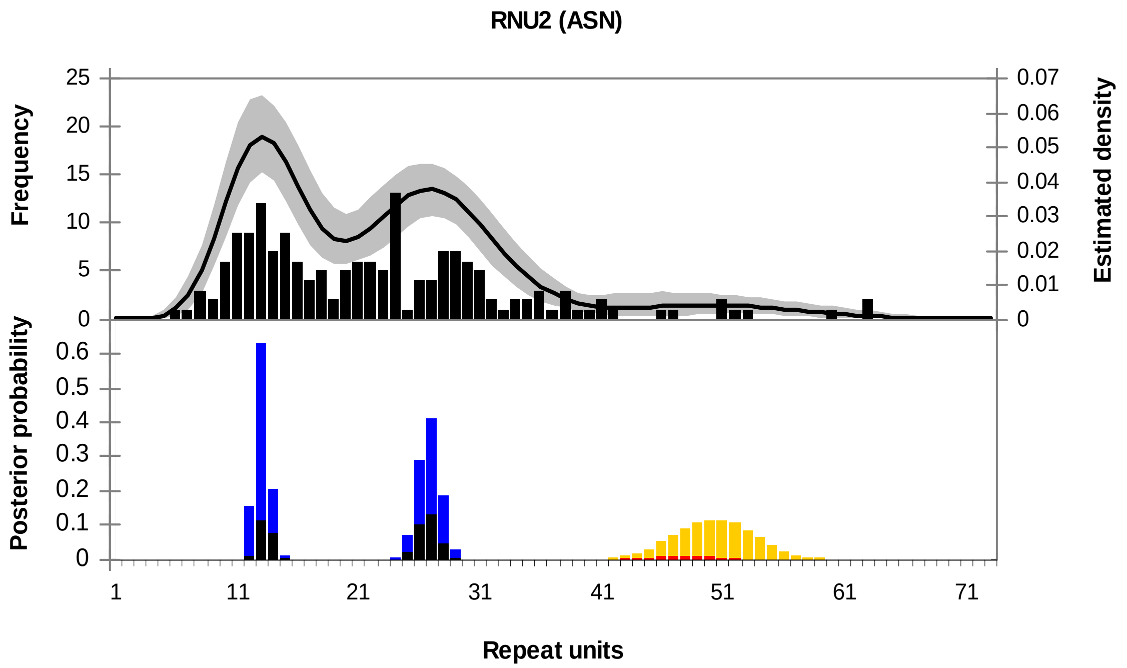

Figure S4a. MSR array size distribution for RNU2 in the Asian population

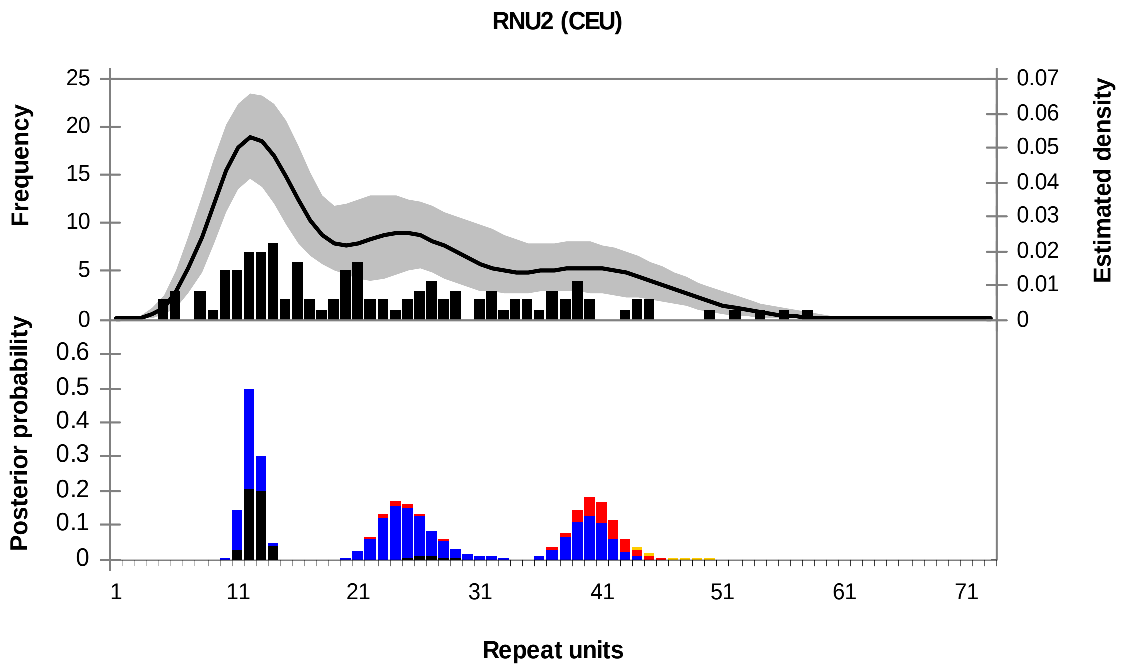

Figure S4b. MSR array size distribution for RNU2 in the Caucasian population

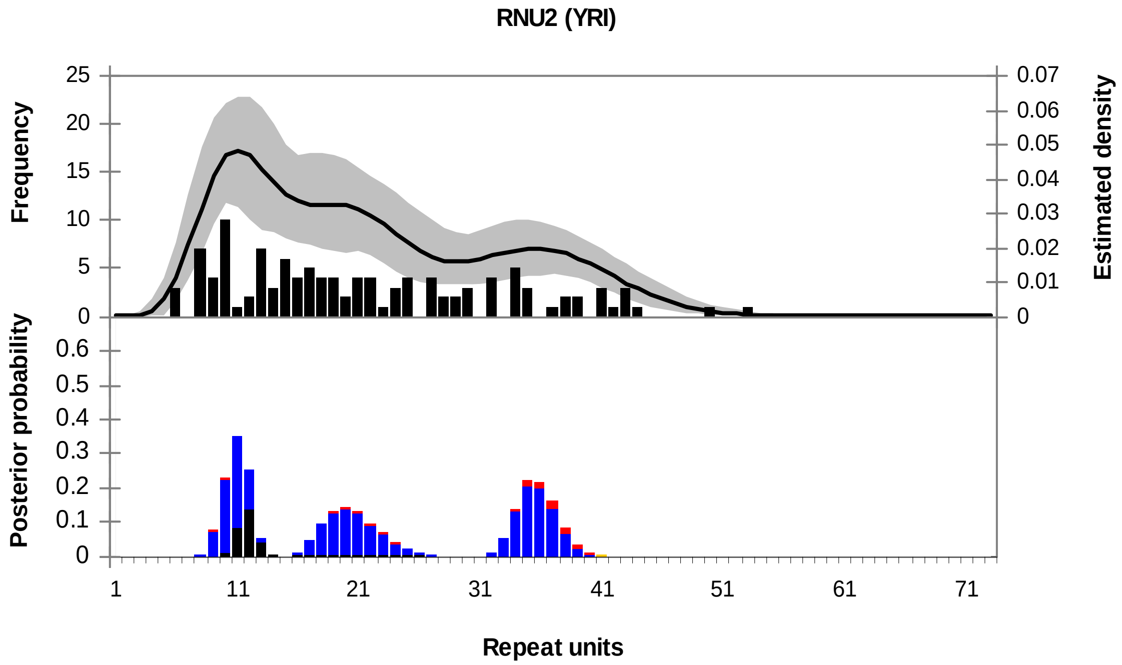

Figure S4c. MSR array size distribution for RNU2 in the African population

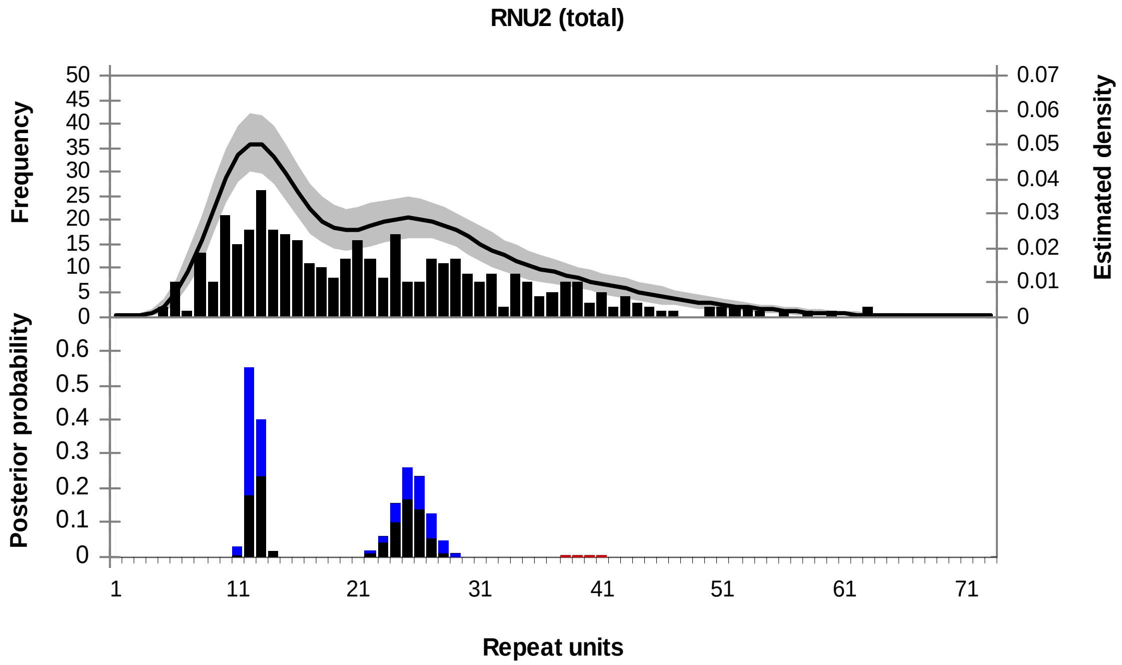

Figure S4d. MSR array size distribution for RNU2 in the total population

Figure S5. MSR array size distributions for DXZ4

Top panel: The histogram shows the size frequencies of the DXZ4 arrays observed in the Asian, Caucasian, African and total population respectively. Based on this frequency distribution of the observed data sample the corresponding estimated density of the unknown underlying distribution was calculated, which is displayed on top of the size histogram as a black line (right y-axis). The 95% posterior interval (i.e., the Bayesian counterpart of the 95% confidence interval) of this estimated density is indicated by the grey area.

Bottom panel: Based on the estimated density of the unknown underlying distribution (and the uncertainty on this estimated density) the presence of modes and their locations was investigated. The graph shows the posterior probability of a mode for each point in the range of the data. The colors indicate the weight of the mode (i.e., the percentage of probability around the mode between the two surrounding minima) in the following order: weight $\geq$ 50% (black), 20% $\leq$ weight < 50% (blue), 10% $\leq$ weight < 20% (red), weight < 10% (yellow).

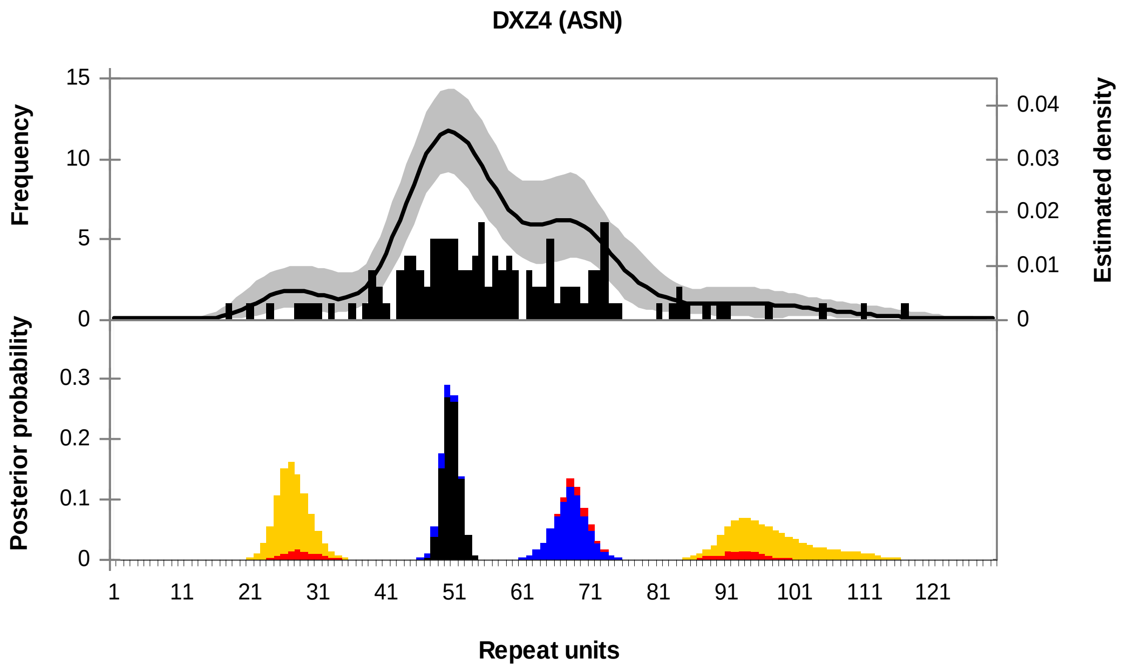

Figure S5a. MSR array size distribution for DXZ4 in the Asian population

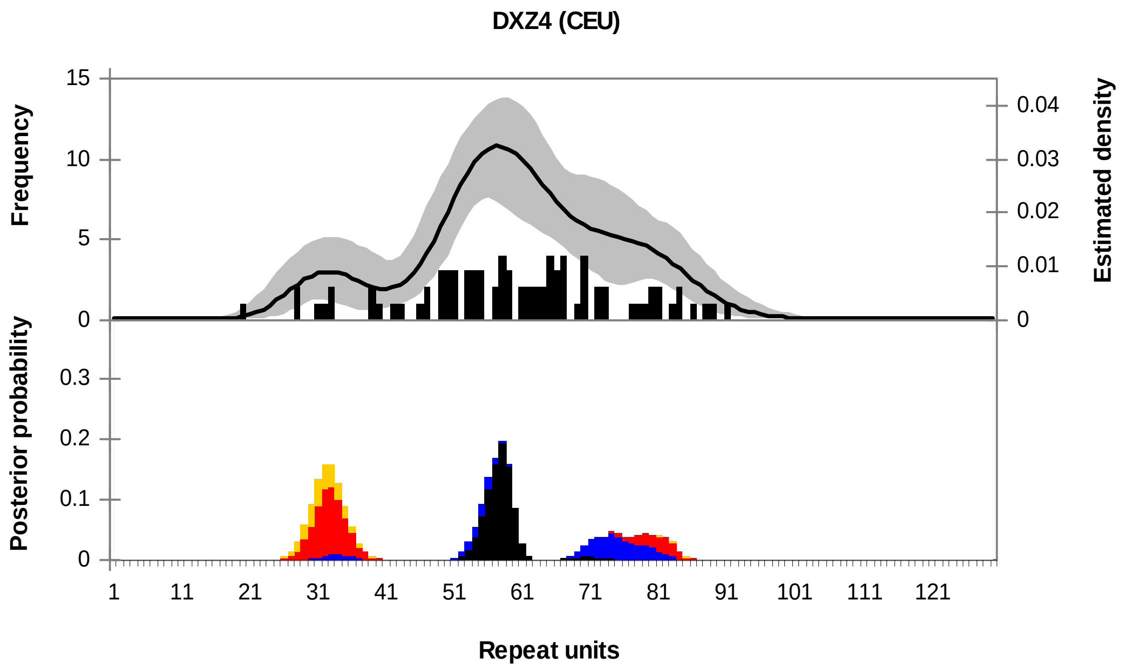

Figure S5b. MSR array size distribution for DXZ4 in the Caucasian population

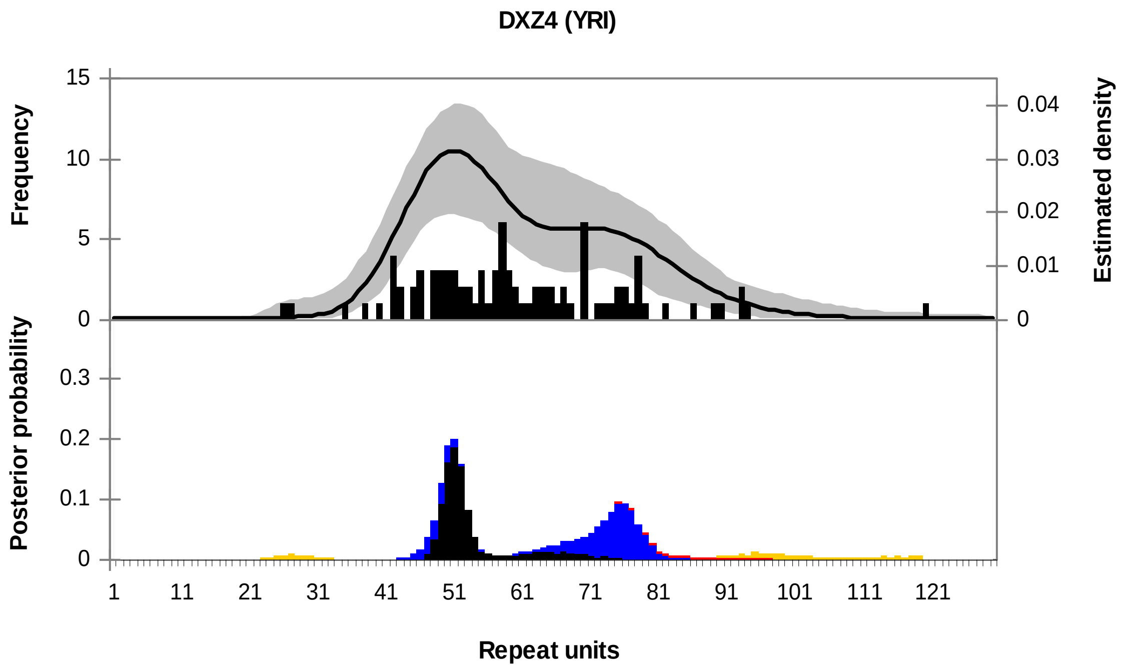

Figure S5c. MSR array size distribution for DXZ4 in the African population

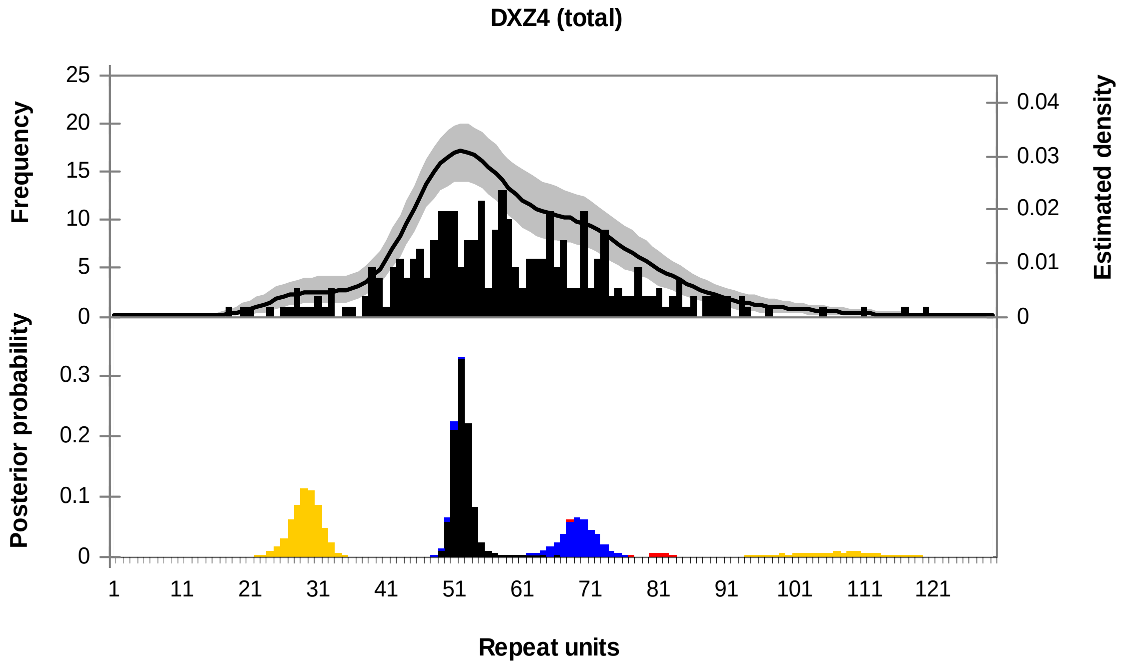

Figure S5d. MSR array size distribution for DXZ4 in the total population

Figure S6. MSR array size distributions for CT47

Top panel: The histogram shows the size frequencies of the CT47 arrays observed in the Asian, Caucasian, African and total population respectively. Based on this frequency distribution of the observed data sample the corresponding estimated density of the unknown underlying distribution was calculated, which is displayed on top of the size histogram as a black line (right y-axis). The 95% posterior interval (i.e., the Bayesian counterpart of the 95% confidence interval) of this estimated density is indicated by the grey area.

Bottom panel: Based on the estimated density of the unknown underlying distribution (and the uncertainty on this estimated density) the presence of modes and their locations was investigated. The graph shows the posterior probability of a mode for each point in the range of the data. The colors indicate the weight of the mode (i.e., the percentage of probability around the mode between the two surrounding minima) in the following order: weight ≥ 50% (black), 20% ≤ weight < 50% (blue), 10% ≤ weight < 20% (red), weight < 10% (yellow).

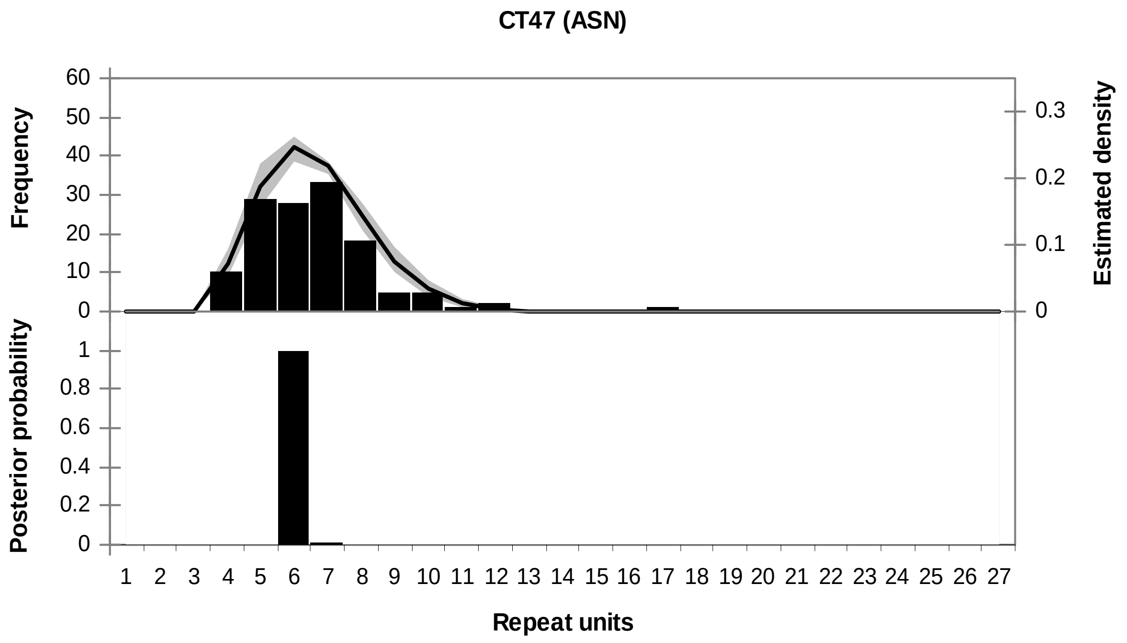

Figure S6a. MSR array size distribution for CT47 in the Asian population

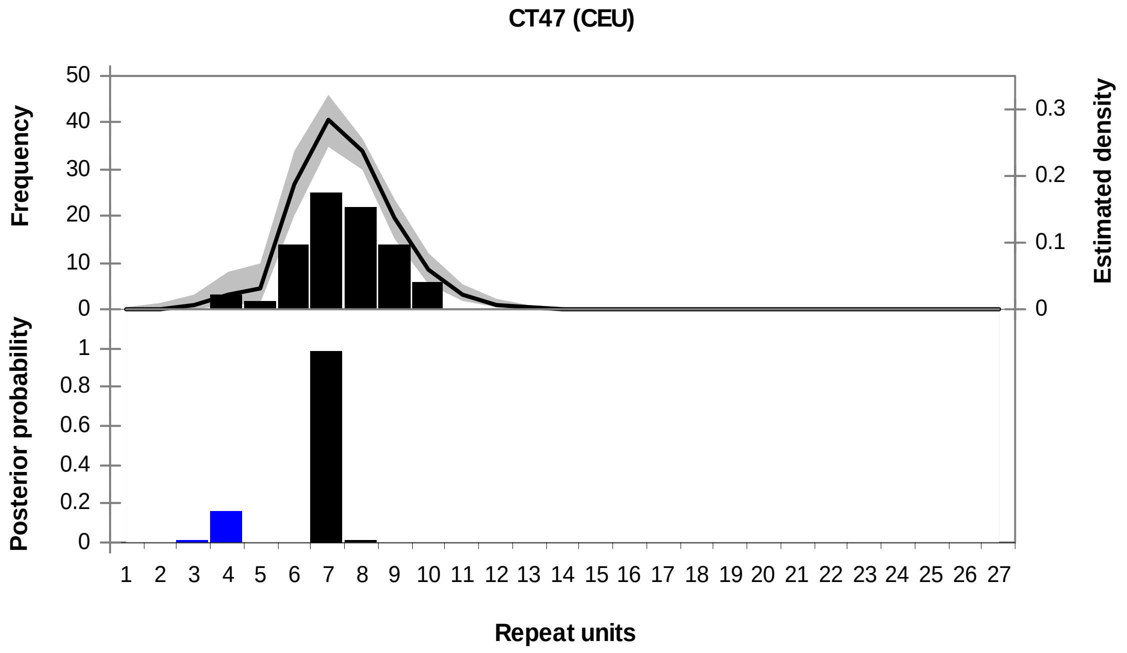

Figure S6b. MSR array size distribution for CT47 in the Caucasian population

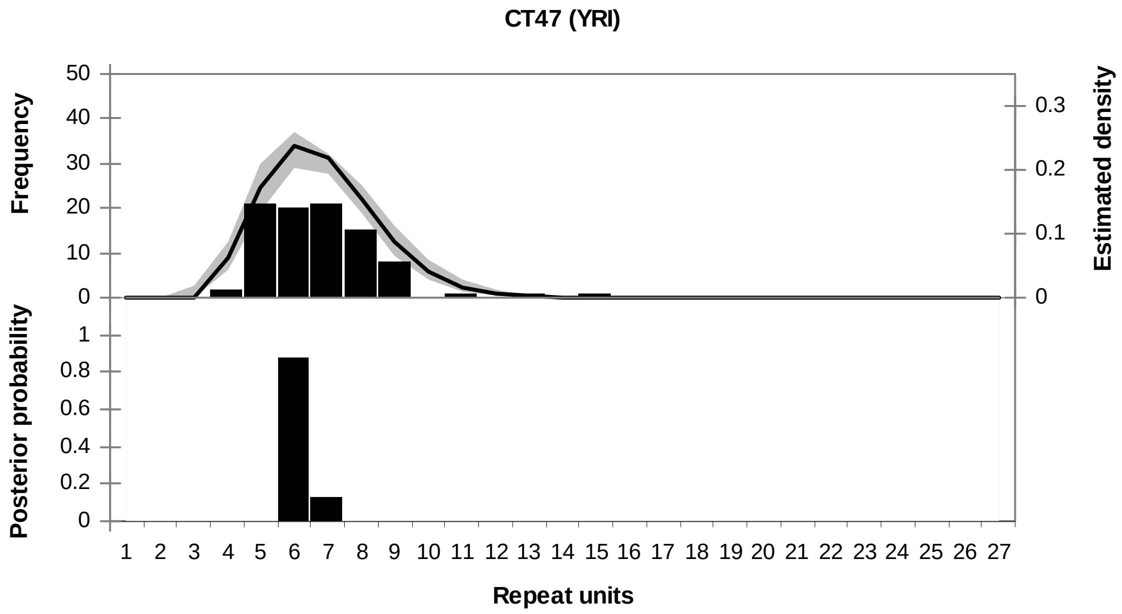

Figure S6c. MSR array size distribution for CT47 in the African population

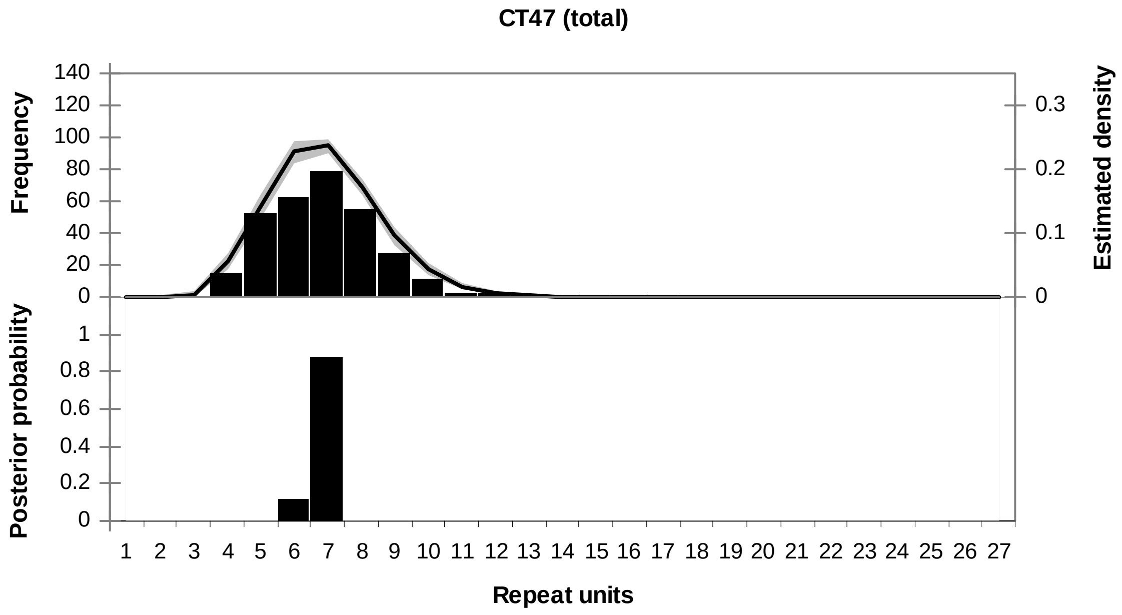

Figure S6d. MSR array size distribution for CT47 in the total population

Figure S7. MSR array size distributions for D4Z4 (4q)

Top panel: The histogram shows the size frequencies of the D4Z4 (4q) arrays observed in the Asian, Caucasian, African and total population respectively. Based on this frequency distribution of the observed data sample the corresponding estimated density of the unknown underlying distribution was calculated, which is displayed on top of the size histogram as a black line (right y-axis). The 95% posterior interval (i.e., the Bayesian counterpart of the 95% confidence interval) of this estimated density is indicated by the grey area.

Bottom panel: Based on the estimated density of the unknown underlying distribution (and the uncertainty on this estimated density) the presence of modes and their locations was investigated. The graph shows the posterior probability of a mode for each point in the range of the data. The colors indicate the weight of the mode (i.e., the percentage of probability around the mode between the two surrounding minima) in the following order: weight ≥ 50% (black), 20% ≤ weight < 50% (blue), 10% ≤ weight < 20% (red), weight < 10% (yellow).

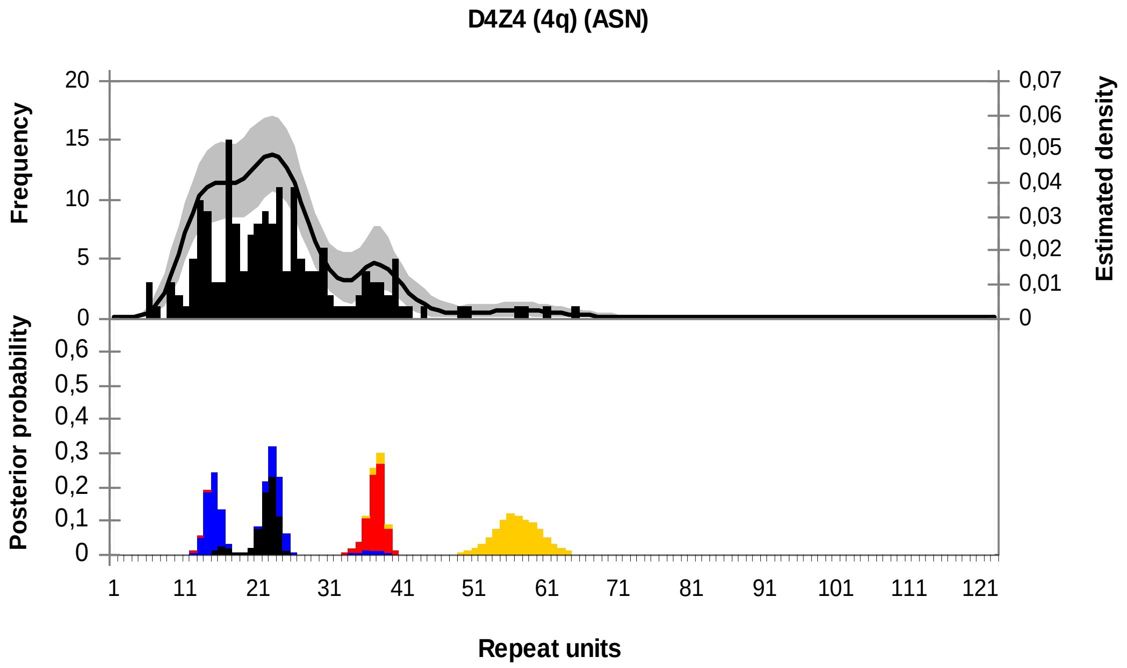

Figure S7a. MSR array size distribution for D4Z4 (4q) in the Asian population

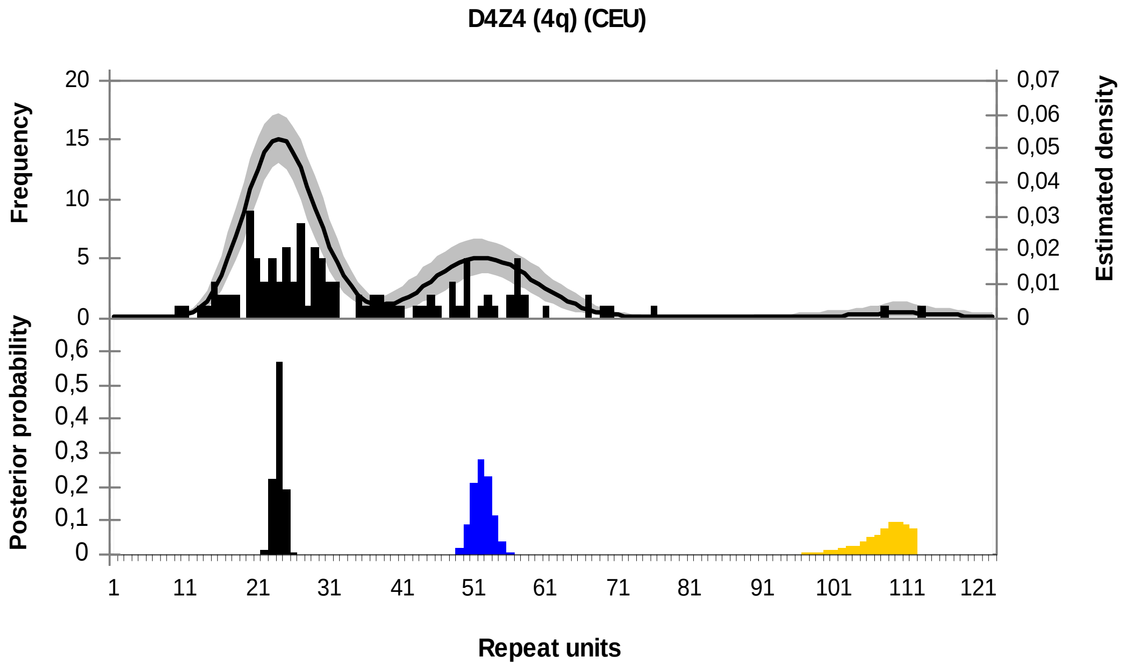

Figure S7b. MSR array size distribution for D4Z4 (4q) in the Caucasian population

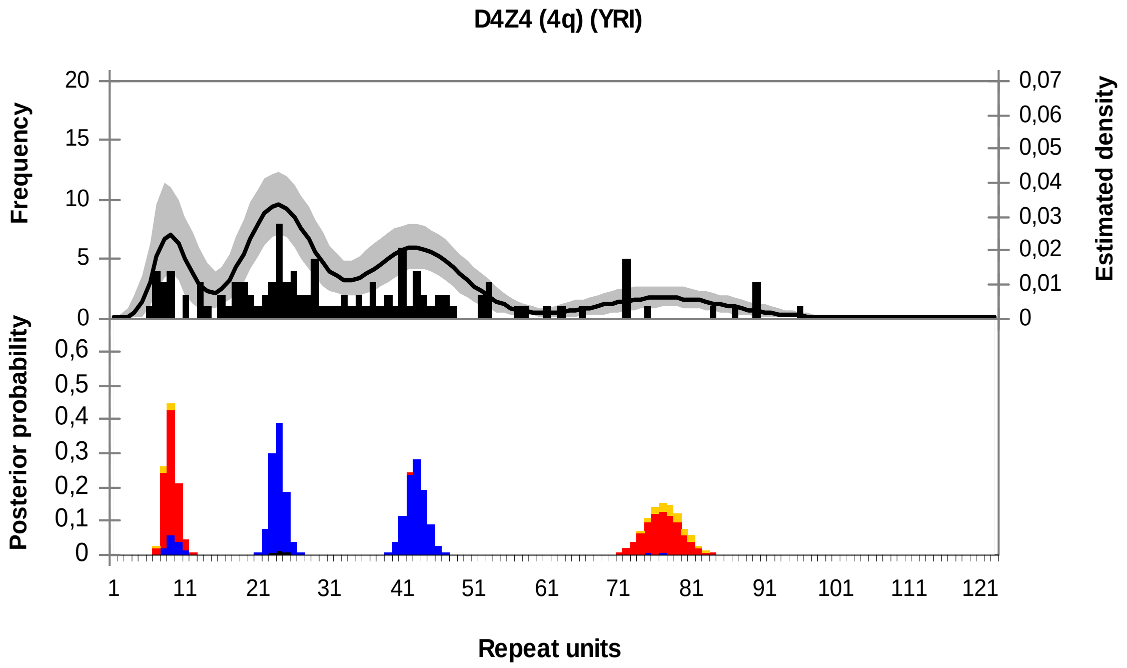

Figure S7c. MSR array size distribution for D4Z4 (4q) in the African population

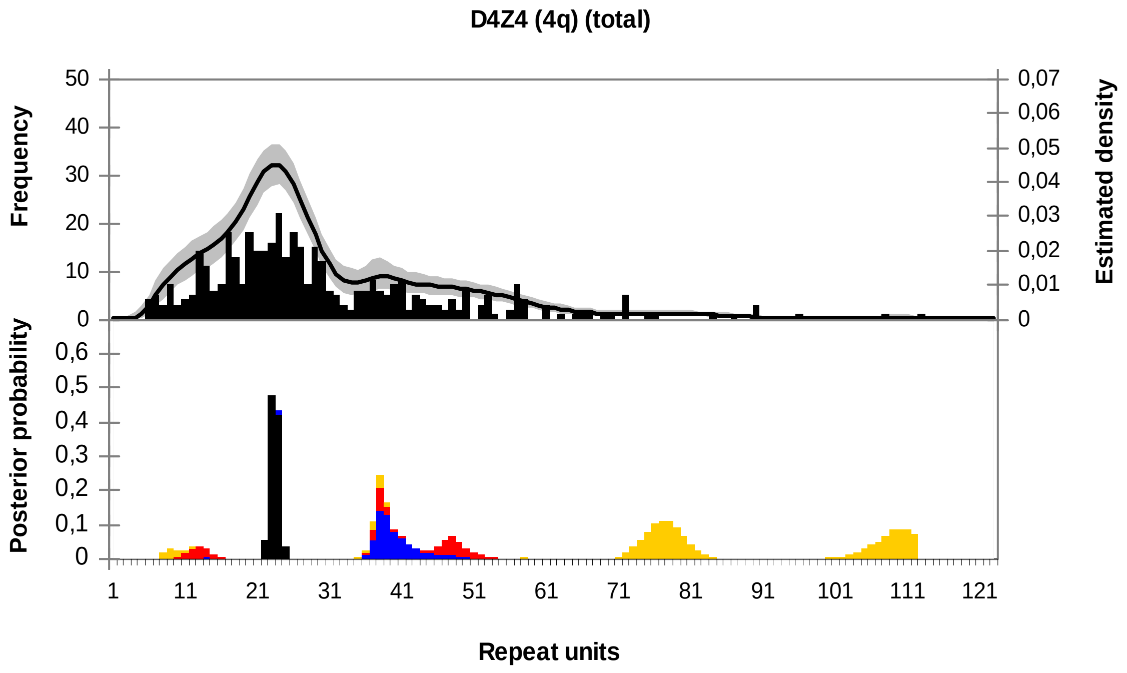

Figure S7d. MSR array size distribution for D4Z4 (4q) in the total population

Figure S8. MSR array size distributions for D4Z4 (10q)

Top panel: The histogram shows the size frequencies of the D4Z4 (10q) arrays observed in the Asian, Caucasian, African and total population respectively. Based on this frequency distribution of the observed data sample the corresponding estimated density of the unknown underlying distribution was calculated, which is displayed on top of the size histogram as a black line (right y-axis). The 95% posterior interval (i.e., the Bayesian counterpart of the 95% confidence interval) of this estimated density is indicated by the grey area.

Bottom panel: Based on the estimated density of the unknown underlying distribution (and the uncertainty on this estimated density) the presence of modes and their locations was investigated. The graph shows the posterior probability of a mode for each point in the range of the data. The colors indicate the weight of the mode (i.e., the percentage of probability around the mode between the two surrounding minima) in the following order: weight ≥ 50% (black), 20% ≤ weight < 50% (blue), 10% ≤ weight < 20% (red), weight < 10% (yellow).

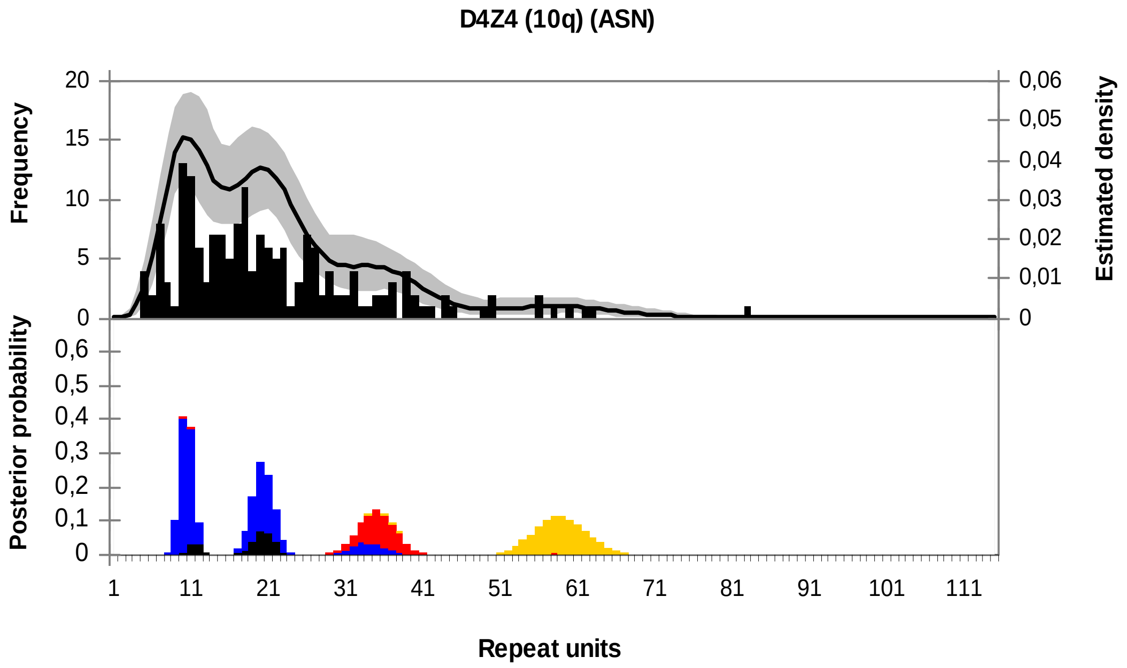

Figure S8a. MSR array size distribution for D4Z4 (10q) in the Asian population

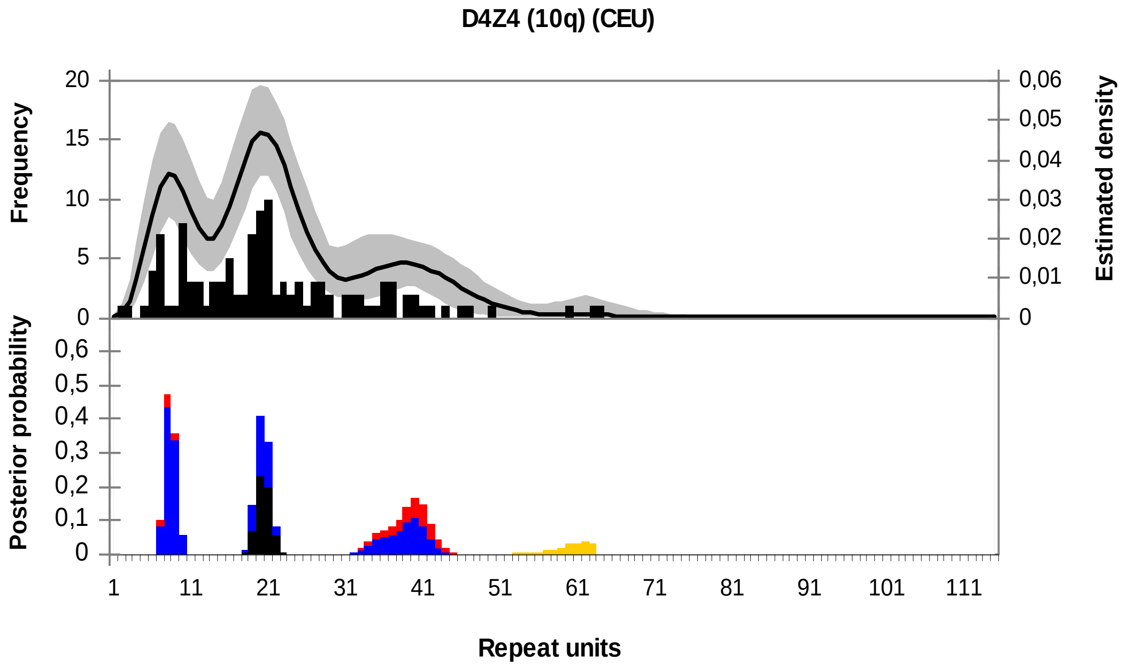

Figure S8b. MSR array size distribution for D4Z4 (10q) in the Caucasian population

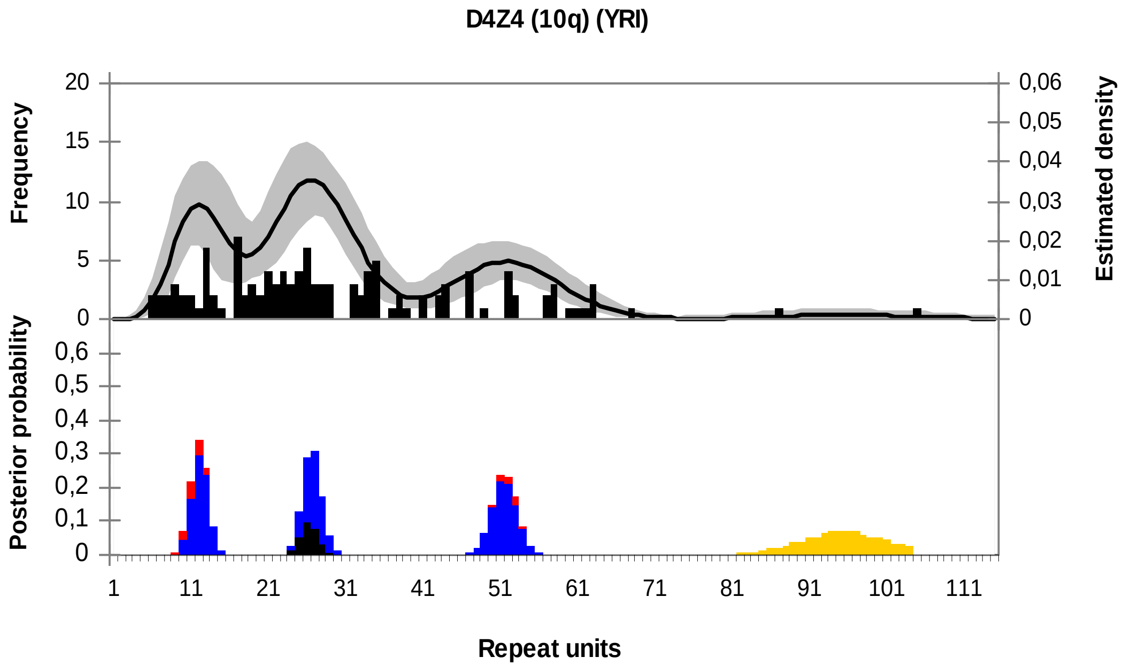

Figure S8c. MSR array size distribution for D4Z4 (10q) in the African population

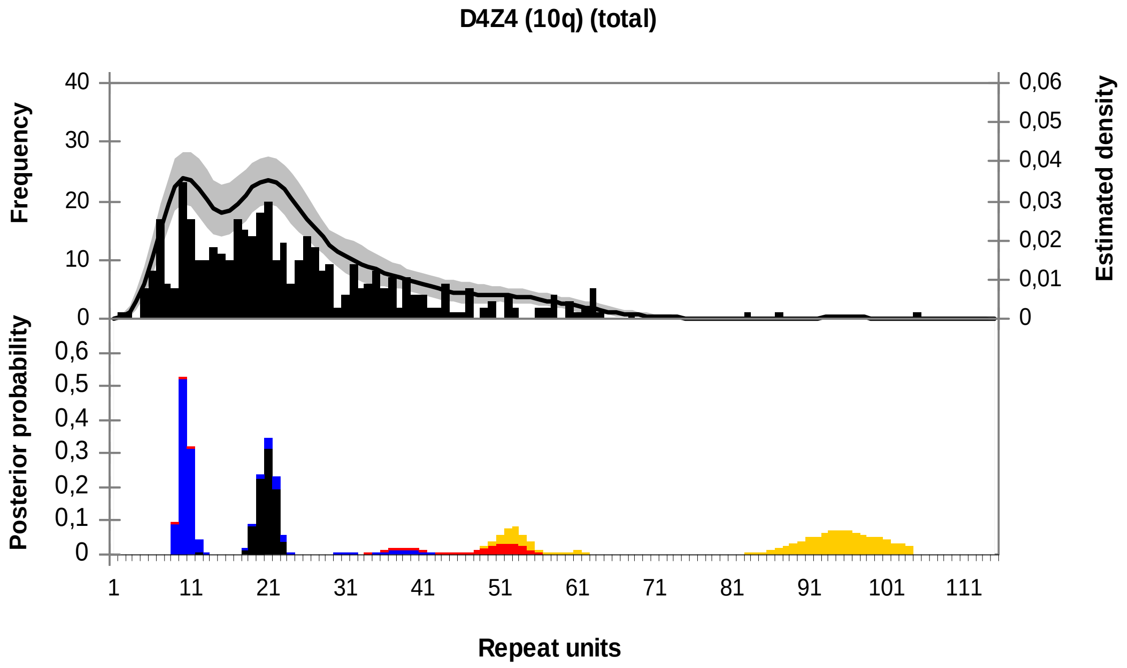

Figure S8d. MSR array size distribution for D4Z4 (10q) in the total population
